# GATA factor-regulated solute carrier ensemble reveals a nucleoside transporter-dependent differentiation mechanism

**DOI:** 10.1371/journal.pgen.1009286

**Published:** 2020-12-28

**Authors:** Nicole M. Zwifelhofer, Xiaoli Cai, Ruiqi Liao, Bin Mao, Daniel J. Conn, Charu Mehta, Sunduz Keles, Yang Xia, Emery H. Bresnick

**Affiliations:** 1 Wisconsin Blood Cancer Research Institute, Department of Cell and Regenerative Biology, Carbone Cancer Center, University of Wisconsin School of Medicine and Public Health, Madison, Wisconsin, United States of America; 2 Department of Biochemistry and Molecular Biology, Graduate School of Biomedical Sciences, University of Texas McGovern Medical School at Houston, Houston, Texas, United States of America; 3 Department of Biostatistics and Medical Informatics, University of Wisconsin School of Medicine and Public Health, Madison, Wisconsin, United States of America; Cincinnati Children’s Hospital Medical Center, UNITED STATES

## Abstract

Developmental-regulatory networks often include large gene families encoding mechanistically-related proteins like G-protein-coupled receptors, zinc finger transcription factors and solute carrier (SLC) transporters. In principle, a common mechanism may confer expression of multiple members integral to a developmental process, or diverse mechanisms may be deployed. Using genetic complementation and enhancer-mutant systems, we analyzed the 456 member SLC family that establishes the small molecule constitution of cells. This analysis identified SLC gene cohorts regulated by GATA1 and/or GATA2 during erythroid differentiation. As >50 SLC genes shared GATA factor regulation, a common mechanism established multiple members of this family. These genes included *Slc29a1* encoding an equilibrative nucleoside transporter (Slc29a1/ENT1) that utilizes adenosine as a preferred substrate. Slc29a1 promoted erythroblast survival and differentiation *ex vivo*. Targeted ablation of murine *Slc29a1* in erythroblasts attenuated erythropoiesis and erythrocyte regeneration in response to acute anemia. Our results reveal a GATA factor-regulated SLC ensemble, with a nucleoside transporter component that promotes erythropoiesis and prevents anemia, and establish a mechanistic link between GATA factor and adenosine mechanisms. We propose that integration of the GATA factor-adenosine circuit with other components of the GATA factor-regulated SLC ensemble establishes the small molecule repertoire required for progenitor cells to efficiently generate erythrocytes.

## Author summary

GATA transcription factors endow blood stem and progenitor cells with activities to produce progeny that transport oxygen to protect cells and tissues, evade pathogens and control physiological processes. GATA factors regulate hundreds of genes, and the actions of these genes mediate important biological functions. While the genes have been documented, many questions remain regarding how the “network” components mediate biological functions. The networks include members of large gene families, and the relationships between the regulation and function of individual family members is not well understood. Analyzing datasets from genetic complementation and enhancer mutant systems revealed that GATA factors regulate an ensemble of membrane transporters termed solute carrier proteins (SLCs), which dictate the small molecule composition of cells. Genetic analyses with Slc29a1, which transports adenosine, revealed its function to promote erythrocyte development, and Slc29a1 attenuated anemia in a mouse model. This study revealed the importance of SLC transporters in GATA factor networks. We propose that the GATA factor-adenosine circuit integrates with other SLCs to establish/maintain the small molecule constitution of progenitor cells as a new mechanism to control blood cell development.

## Introduction

As a process that broadly informs stem cell biology, hematopoietic stem cells produce diverse progenitor cells that differentiate into blood cells, ensuring physiological homeostasis and the capacity to respond to stress [1–3]. Lineage-committed progenitor cells undergo drastic molecular and cellular transitions to generate blood cell types with overtly different phenotypes and functions. For example, erythroid progenitor cells differentiate into precursor cells that progressively mature into enucleated reticulocytes and erythrocytes [4]. In pathological states, such as anemia resulting from acute blood loss, a “stress erythropoiesis” mechanism is deployed to accelerate erythrocyte regeneration and oxygen delivery, thereby protecting cells and tissues [5, 6].

In addition to informing stem cell biology, hematopoiesis represents a powerful system for addressing fundamental problems in molecular biology and genetics, including how complex genetic, protein and small molecule networks control cellular differentiation. The GATA transcription factors GATA2 and GATA1 instigate genetic networks in hematopoietic stem and progenitor cells (HSPCs), erythroid precursor cells and erythroblast progeny [7]. GATA2 is expressed in erythroid precursor cells, and as GATA1 increases, it acquires the capacity to repress *Gata2* transcription [8]. This GATA switch often decreases or increases GATA factor target gene transcription and impacts hundreds to thousands of proteins in the erythroblast proteome [9–12]. The target genes include members of large gene families, e.g. G-protein-coupled receptors, zinc finger transcription factors and solute carrier (SLC) transporters.

In a mechanism in which multiple members of a large gene family are integral to a developmental process, it is instructive to compare and contrast the regulation and function of the family members. Contrasting with the six-member mammalian GATA factor family [1, 7, 13–16], a large gene family may contain hundreds of members. In principle, a common mechanism may establish expression of multiple family members, or an array of mechanisms may be deployed. To address this problem in the context of networks governing erythropoiesis, we analyzed the >450 SLC transporters that dictate the small molecule repertoire of cells [17]. Several SLCs are implicated in erythroid biology [18–24] including the GATA1-induced gene *Slc4a1* [25] encoding an anion transporter in erythroblasts and erythrocytes [26]. GATA1 instigates a zinc transporter switch, involving the importer Slc39a8 and exporter Slc30a1, that controls intracellular zinc and erythroid differentiation [12]. We reasoned that an SLC transporter cohort establishes/maintains the erythroblast small molecule repertoire as a vital element in GATA factor-dependent networks. Identifying essential SLCs will enable building of integrative models to explain how extracellular stimuli utilize small molecules to orchestrate cellular functions.

Given the large number of SLC transporters not studied in hematopoiesis, we evaluated GATA1- and GATA2-regulated genes in genetic rescue and enhancer-mutant systems, respectively, to identify GATA factor-regulated SLCs. GATA1 and GATA2 co-regulated >50 SLCs including amino acid, metal and nucleoside/nucleotide transporters. Embedded within this cohort are eight SLCs, including the equilibrative nucleoside transporter 1 (Slc29a1/ENT1), that share GATA factor occupancy at predicted intronic enhancer regions containing predicted *cis*-regulatory elements, suggesting direct transcriptional regulation. Loss-of-function studies indicated that an Slc29a1-dependent mechanism promotes erythroblast survival and differentiation and attenuates anemia in an acute anemia mouse model. As Slc29a1 transports adenosine, and GATA factors induced adenosine kinase, which converts adenosine to AMP, GATA factor-dependent networks contain an adenosine circuit that is expected to control vital biological processes.

## Results

### A GATA1- and GATA2-regulated solute carrier gene ensemble

**Fig 1:**
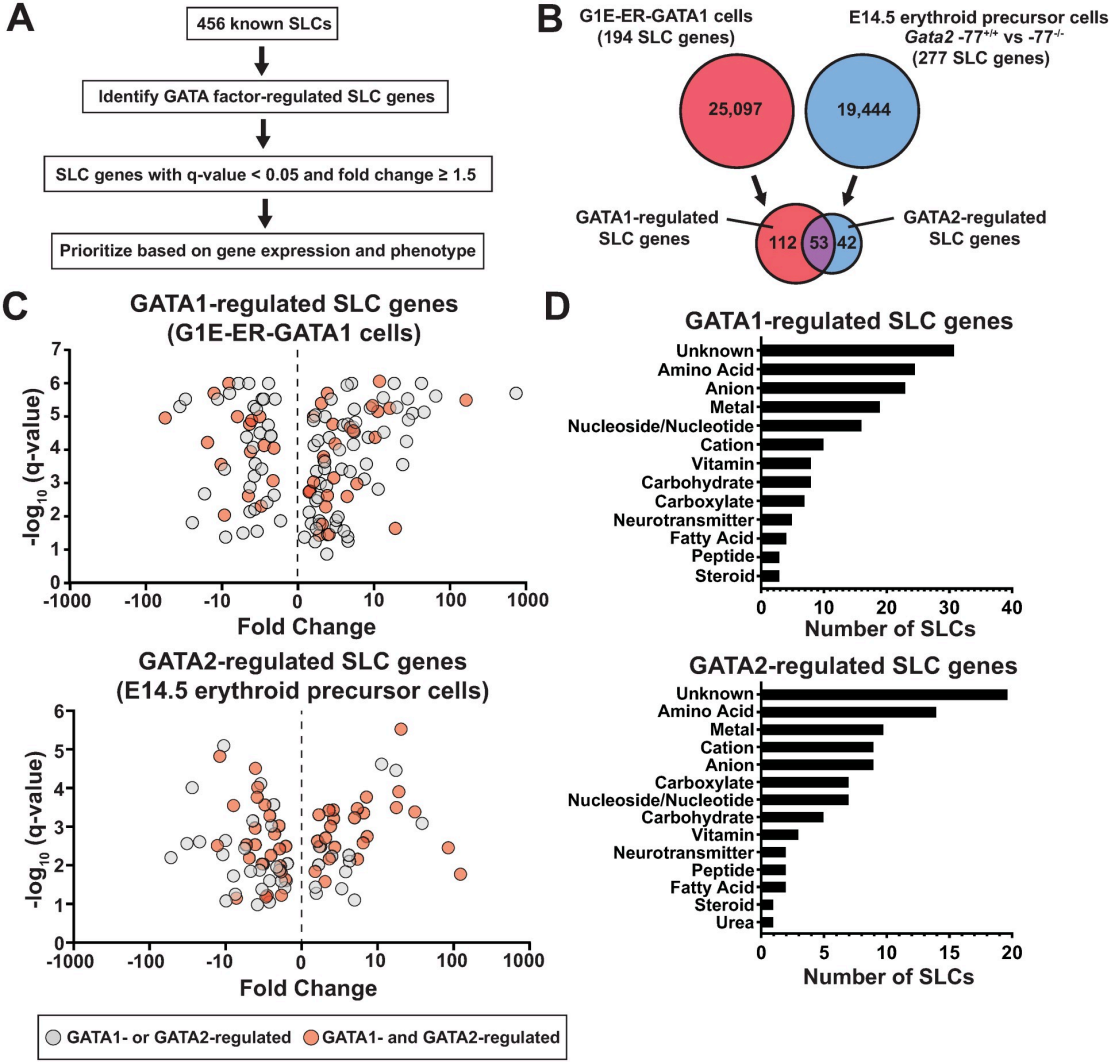
GATA factor-regulated SLC transporter gene ensemble. (A) Prioritization strategy used to identify GATA1- and GATA2-regulated SLC genes. GATA1-regulated genes were identified by RNA-seq with a G1E-ER-GATA1 genetic complementation assay in GATA1-null G1E cells expressing ER-GATA1 [27]. GATA2-regulated genes were identified by RNA-seq by comparing gene expression *Gata2*–77^+/+^ and -77^-/-^ enhancer mutant erythroid precursor cells [31]. The scoring system utilized existing gene expression and phenotype information. (B) The Venn diagram depicts GATA1- and/or GATA2-regulated SLCs. (C) The volcano plots depict statistically significant RNA-Seq data of GATA1- (top) or -77 enhancer-regulated (bottom) SLC genes (≥ 1.5 fold). The red circles denote GATA1- and GATA2-regulated SLC genes. (D) The bar graphs depict small molecules known to be transported by GATA1-regulated (top) and -77 enhancer-regulated (bottom) SLCs.

Since SLCs regulate a wide spectrum of intracellular small molecules, and very little is known about how GATA factors impact small molecule-dependent mechanisms, we conducted an analysis to identify all GATA1- and GATA2-regulated SLC genes in erythroblasts. We used our RNA-seq dataset [27] from the GATA1-null G1E-ER-GATA1 cell genetic rescue system [28] to identify GATA1-regulated SLC genes (Fig 1A). Treatment of cells with β-estradiol (48 hours) activates a conditional GATA1 allele (ER-GATA1) stably expressed in proerythroblast-like G1E cells, thus inducing or repressing GATA1 target genes and stimulating erythroid differentiation [25]. This analysis revealed 165 GATA1-regulated SLC genes (≥1.5 fold) (Fig 1B).

GATA2 is expressed in erythroid precursor cells prior to GATA1 [29]. As GATA1 accumulates, GATA1 acquires the capacity to repress *Gata2* transcription [8]. GATA2 functions through the *Gata2*–77 enhancer (-77) to confer *Gata2* transcription in myelo-erythroid progenitor [30] and erythroid precursor cells [31]. In these cells, -77 enhancer deletion reduces *Gata2* expression and protein levels ~80%, and GATA2 re-expression rescues molecular/cellular phenotypes [30, 32]. To determine if GATA2 regulates an SLC gene cohort resembling or distinct from the GATA1-regulated cohort, we used our RNA-seq dataset that analyzed differentially expressed genes in *Gata2*–77^+/+^ vs. -77^-/-^ primary murine fetal liver erythroid precursor cells [31]. This analysis identified 95 SLC genes regulated by GATA2 ≥1.5 fold, and 53 SLC genes were GATA1- and GATA2-regulated (Fig 1B).

**Fig 2:**
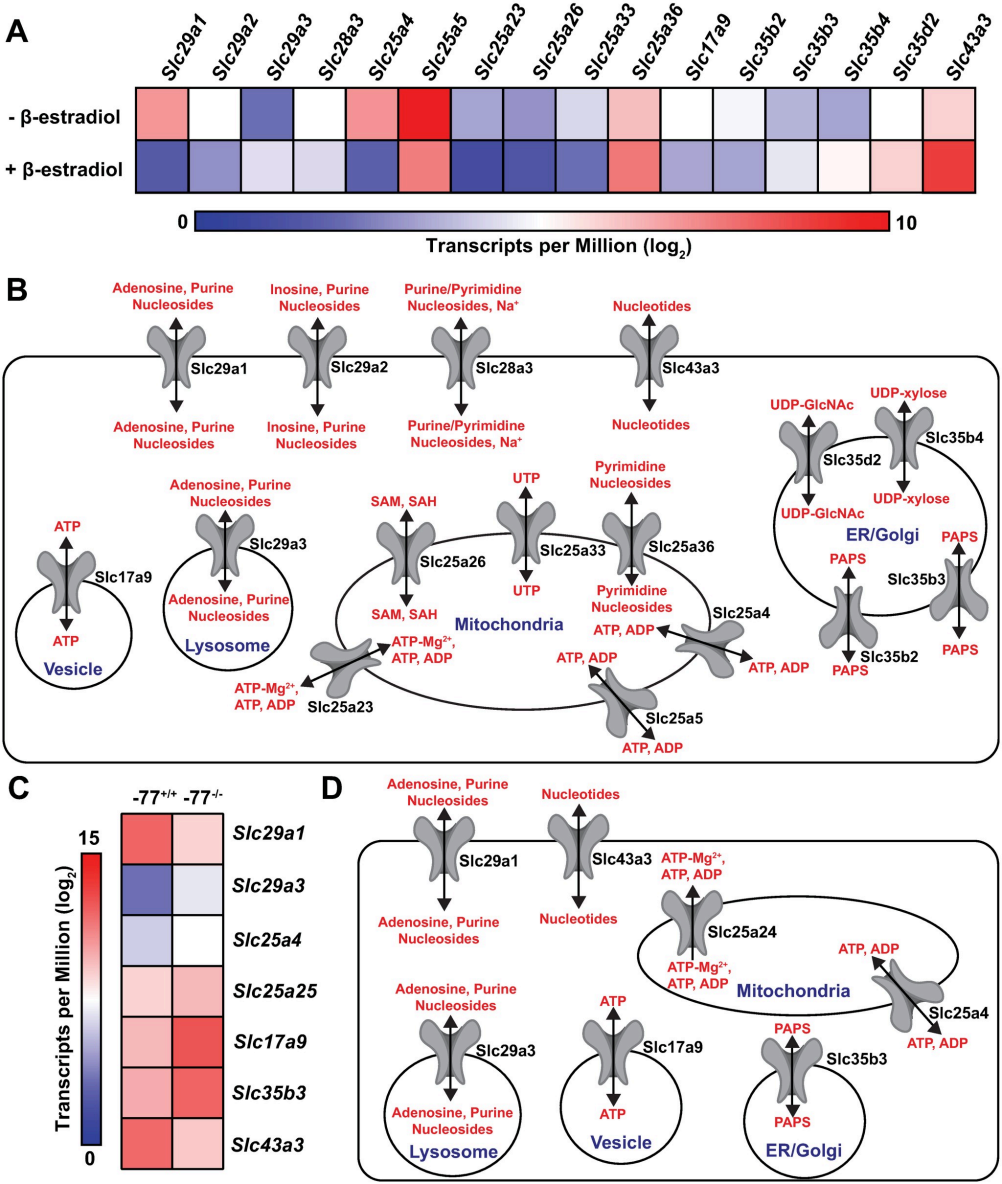
GATA factor-regulated nucleoside/nucleotide SLC transporter cohort. (A) The heatmap depicts GATA1-regulated nucleoside/nucleotide SLC mRNAs derived from comparison of G1E-ER-GATA1 cell RNA-seq data (untreated vs. 48 hour β-estradiol-treated cells) [27]. (B) Schematic representation of established substrates and cellular/subcellular localizations of the GATA1-regulated SLC nucleoside/nucleotide transporters. (C) The heatmap depicts GATA2-regulated nucleoside/nucleotide SLC mRNAs from comparison of *Gata2*–77 enhancer-mutant vs. wild-type erythroid precursor RNA-seq data [31]. (D) Schematic representation of established substrates and cellular/subcellular localizations of the GATA2-regulated SLC nucleoside/nucleotide transporters.

The GATA factor-regulated SLC genes were parsed based on which GATA factor they were regulated by and whether they were activated or repressed. GATA1 activated and repressed 103 and 62 SLC genes, whereas GATA2 activated and repressed 39 and 56, respectively (Fig 1C). Considering the established substrates transported by these SLCs (Fig 1D), amino acid (24 and 16, respectively) (S1A and S1B Fig), metal (18 and 12, respectively) (S1C and S1D Fig) and nucleoside/nucleotide transporters (16 and 7, respectively) were well represented in GATA1- and GATA2-regulated SLC cohorts. Amino acid, metal and nucleoside/nucleotide transporters were enriched in the GATA factor-co-regulated cohort relative to their contribution to the >450 SLC gene ensemble (p = 0.04, 0.03 and 0.04, respectively) (S2 Fig). Of the 16 GATA1-regulated nucleoside transporters, seven were activated, and nine were repressed (Fig 2A). This cohort transports diverse nucleosides and/or nucleotides, including purine and pyrimidine nucleosides, nucleotides (e.g. ATP/ADP, UTP/UDP, GTP/GDP) and substrates of the nucleoside/nucleotide sugar transporter family Slc35, including uridine diphosphate N-acetylglucosamine (UDP-GlcNAc), 3'-phosphoadenosine-5'-phosphosulfate (PAPS), and UDP-xylose. Among the 16 transporters, 12 are intracellular, six localize to mitochondria, and four to ER/Golgi (Fig 2B). The seven GATA2-regulated nucleoside and/or nucleotide transporters revealed two GATA2-activated and five GATA2-repressed SLCs (Fig 2C). Among this cohort, six (*Slc29a1*, *Slc29a3*, *Slc25a4*, *Slc17a9*, *Slc35b3* and *Slc43a3*) of the seven were GATA1- and GATA2-regulated. Five of the seven SLCs localize to intracellular compartments and transport diverse purine and pyrimidine nucleosides, nucleotides, and nucleoside/nucleotide sugars (Fig 2D). Since nutrient deficiencies (i.e. iron, folate, thiamine, etc) have potential to cause anemia, we investigated whether SLCs with blood-disease causing mutations were GATA-regulated. Within the GATA1-regulated vitamin transporter cohort, GATA1 upregulated the mitochondrial and plasma membrane-bound thiamine transporter Slc19a2/Thtr1 (S1A Fig). Thiamine is required for heme biosynthesis and human *SLC19A2* mutations cause thiamine-responsive megaloblastic anemia syndrome [33–35].

**Fig 3:**
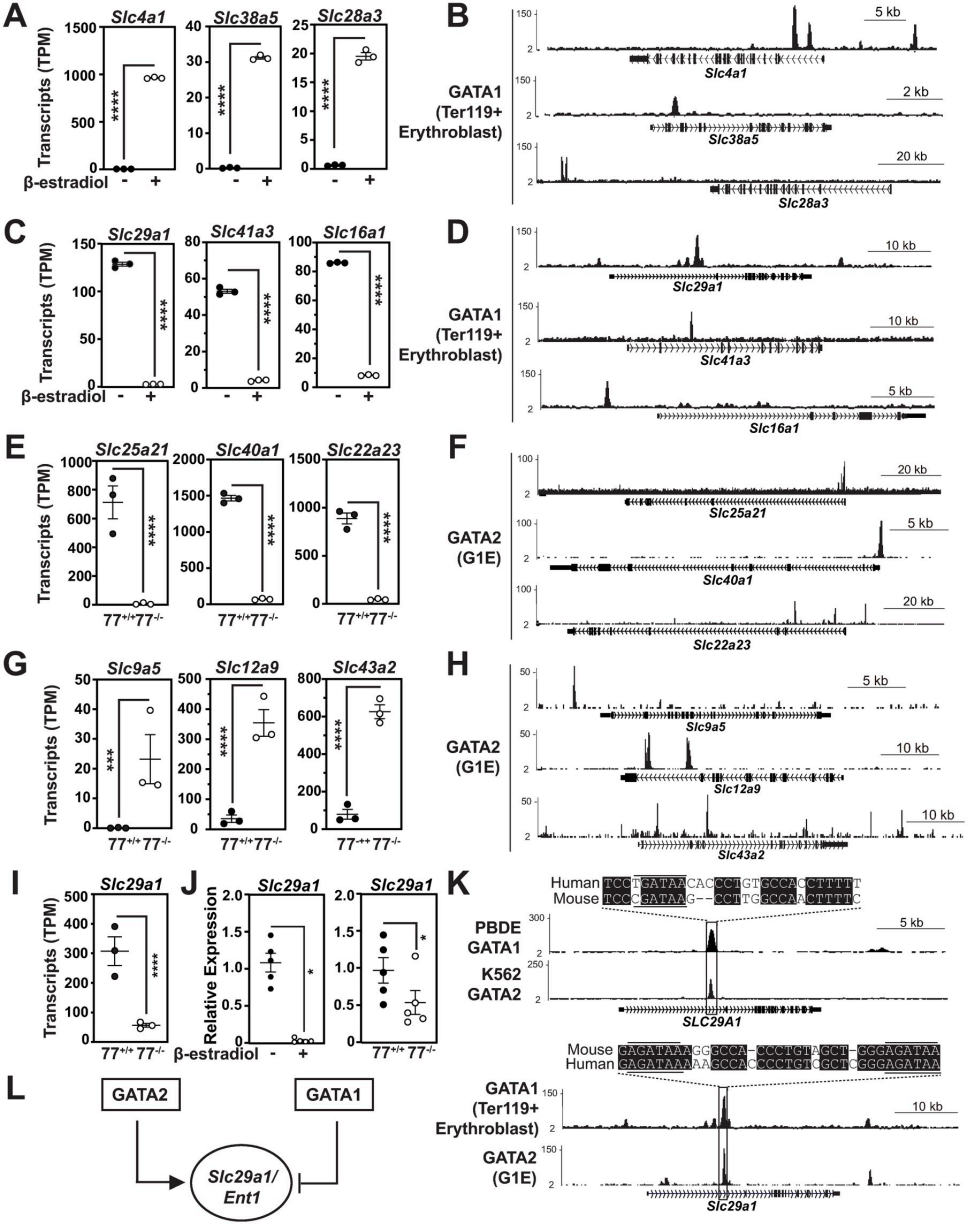
GATA1 and GATA2 chromatin occupancy at SLC transporter genes. (A) GATA1 activation of *Slc4a1*, *Slc38a5*, and *Slc28a3* based on comparison of expression in control vs. β-estradiol-treated (48 h) G1E-ER-GATA1 cells. (B) ChIP-seq profiles demonstrating GATA1 occupancy at *Slc4a1*, *Slc38a5* and *Slc28a3* loci in Ter119+ erythroblasts (GEO GSE30142) [40]. (C) GATA1 repression of *Slc29a1*, *Slc41a3*, and *Slc16a1* based on comparison of expression in control vs. β-estradiol-treated (48 h) G1E-ER-GATA1 cells. (D) ChIP-seq profiles demonstrating GATA1 occupancy at *Slc29a1*, *Slc41a3*, and *Slc16a1* loci in Ter119+ erythroblasts (GEO GSE30142) [40]. (E) GATA2 activation of *Slc25a21*, *Slc40a1*, and *Slc22a23* based on comparison of expression in *Gata2*–77^+/+^ vs. -77^-/-^ primary erythroid precursor cells. (F) ChIP-seq profiles demonstrating GATA2 occupancy at *Slc25a21*, *Slc40a1* and *Slc22a23* in G1E proerythroblasts (GEO GSE29196) [38]. (G) GATA2 repression of *Slc9a5*, *Slc12a9* and *Slc43a2*, based on comparison of expression in *Gata2*–77^+/+^ vs. -77^-/-^ erythroblasts. (H) ChIP-Seq profiles demonstrating GATA2 occupancy at *Slc9a5*, *Slc12a9* and *Slc43a2* in G1E proerythroblasts (GEO GSE29196) [38]. (I) *Slc29a1* mRNA in *Gata2*–77^+/+^ vs. -77^-/-^ erythroblasts. (J) qRT-PCR analysis of *Slc29a1* mRNA in untreated vs. G1E-ER-GATA1 cells treated with β-estradiol for 48 hours (left) and in *Gata2*–77^+/+^ vs. -77^-/-^ erythroid precursor cells (right). (K) ChIP-seq profiles depicting GATA1 and GATA2 occupancy at the human (top) and mouse (bottom) *Slc29a1* in human peripheral blood-derived erythroblasts (GEO GSE32491) [47], K562 cells (GEO GSE18829) [10] murine Ter119^+^ erythroblasts (GEO GSE30142) [40] and G1E proerythroblasts (GEO GSE29196) [38]. Underlined sequences indicate WGATAR motifs. Conserved mouse and human sequences are represented by white text with black background. (L) Model depicting opposing regulation of *Slc29a1* by GATA1 and GATA2. Scatter plots represent means ± SEM. TPM; Transcripts per million (*n* = 3). mRNA levels were normalized to 18S rRNA (*n* = 5). Statistical analysis was carried out with an unpaired 2-tailed Student’s *t* test. * *p* < 0.05, **** *p* < 0.0001.

To prioritize GATA factor-regulated SLC genes for functional analysis, we identified those activated or repressed to the greatest extent and asked if GATA1 or GATA2 occupy chromatin at these loci. Among the top 10 GATA1-activated SLC genes from the G1E-ER-GATA1 RNA-seq analysis, *Slc4a1* (396 fold), *Slc38a5* (182 fold) and *Slc28a3* (32.1 fold) were the top three GATA1-activated/GATA1-occupied SLC genes in Ter119+ erythroblasts (GEO GSE30142) (Fig 3A–3B). Within the top 10 GATA1-repressed SLC genes, *Slc29a1* (40.9 fold), *Slc41a3* (10.2 fold), and *Slc16a1* (8.4 fold) were the top three GATA1-repressed/GATA1-occupied SLC genes in murine Ter119+ erythroblasts supporting a direct transcriptional mechanism (Fig 3C–3D).

To identify GATA2-regulated SLCs, we mined RNA-seq data from -77^+/+^ vs. -77^-/-^ primary erythroid precursors. The top 10 GATA2-activated/GATA2-occupied (in undifferentiated G1E proerythroblasts; GEO GSE29196) SLCs included *Slc25a21* (87.0 fold), *Slc40a1* (20.4 fold) and *Slc22a23* (19.1 fold) (Fig 3E–3F). The top 10 GATA2-repressed/GATA2-occupied SLCs included *Slc9a5* (617 fold), *Slc12a9* (10 fold) and *Slc43a2* (7.9 fold) (Fig 3G–3H).

Using a prioritization strategy involving the magnitude of GATA factor regulation and reported hematopoietic-relevant human or mouse phenotypes, we delimited an SLC cohort for further study. One of the top 10 GATA1-repressed SLC genes within this cohort was *Slc29a1*, which encodes the nucleoside transporter Slc29a1/ENT1 that mediates uptake of adenosine and certain chemotherapeutic drugs [36]. The -77^+/+^ vs. -77^-/-^ erythroid precursor RNA-seq data [31] revealed GATA2 activation of *Slc29a1* expression 5.4 fold (Fig 3I). By qRT-PCR, GATA1 decreased and -77 increased *Slc29a1* expression 26 and 1.9 fold, respectively (Fig 3J). Both GATA1 (GEO GSE32491) and GATA2 (GEO GSE18829) occupied the human *SLC29A1* locus at a site harboring a consensus GATA motif. ChIP-seq with primary murine Ter119^+^ erythroblasts (GEO GSE30142) or undifferentiated G1E proerythroblasts (GEO GSE29196) revealed endogenous GATA1 and GATA2 occupancy, respectively, within the *Slc29a1* first intron harboring two consensus GATA motifs (Fig 3K). In aggregate, these results demonstrate that GATA2 and GATA1 activate and repress *Slc29a1*, respectively (Fig 3L), and regulate an SLC ensemble enriched in amino acid, metal and nucleoside/nucleotide transporters.

Using published RNA-seq data (GEO GSE53983) [37], we analyzed expression of this SLC gene cohort upon erythroid differentiation of primary human CD34^+^ cells. *SLC4A1* and *SLC28A3* were upregulated, consistent with their GATA1 induction in G1E-ER-GATA1 cells (S3A Fig). *SLC29A1* and *SLC41A3* were downregulated upon differentiation, consistent with GATA1 repression in G1E-ER-GATA1 cells (S3B Fig). *SLC25A21*, *SLC40A1*, *SLC22A23*, *SLC12A9*, and *SLC43A2* were differentially regulated in the human CD34^+^ cell vs. the -77^-/-^ murine erythroid precursor cell systems (S3C and S3D Fig).

**Fig 4:**
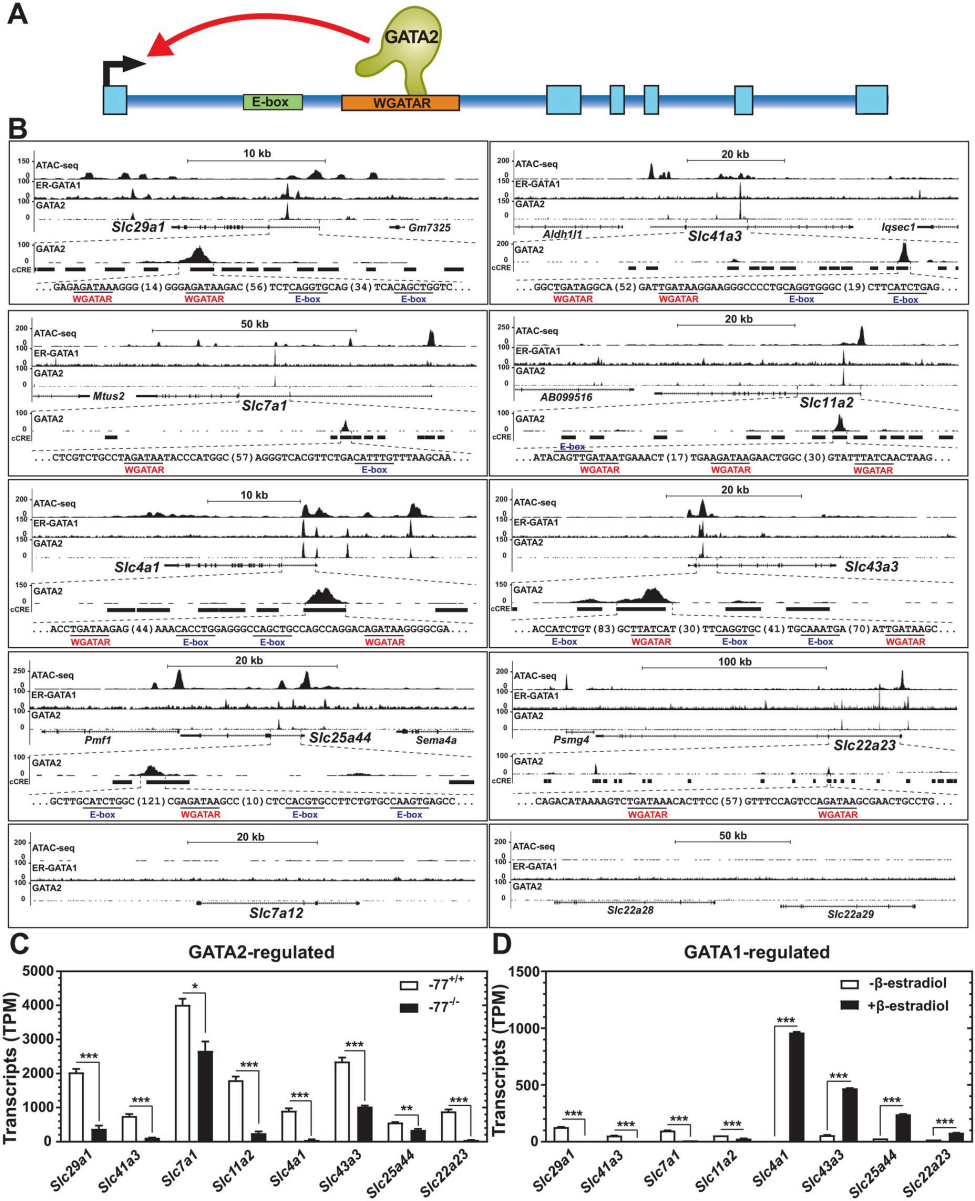
An SLC cohort with GATA factor occupancy at predicted intronic enhancers. (A) Model depicting direct GATA2 regulation via intronic enhancers with WGATAR and E-box motifs. (B) ATAC-seq (GEO GSE114996) [39] and ChIP-seq profiles (GEO GSE30142, GEO GSE29196) [38, 40] of GATA1/2 co-regulated SLC genes (*Slc29a1*, *Slc41a3*, *Slc7a1*, *Slc11a2*, *Slc4a1*, *Slc43a3*, *Slc25a44*, and *Slc22a23*), and control genes (*Slc7a12*, *Slc22a28* and *Slc22a29*) in G1E or G1E-ER-GATA1 cells. The GATA2 ChIP-seq peak is enlarged to illustrate candidate *cis*-regulatory elements (black bars)[41], WGATAR motifs (underlined, red text), and E-Box motifs (underlined, blue text). (C) RNA abundance measured by RNA-seq with *Gata2*–77^-/-^ and -77^+/+^ erythroblasts (*n* = 3). (D) RNA abundance measured by RNA-seq with untreated or 48-hour β-estradiol-treated G1E-ER-GATA1 cells (*n* = 3).

We parsed the GATA2/GATA1-co-regulated SLC cohort into four groups of GATA2-upregulated/GATA1-upregulated, GATA2-upregulated/GATA1-downregulated, GATA2-downregulated/GATA1-upregulated, and GATA2-downregulated/GATA1-downregulated (S1 Table). We analyzed GATA2 (in G1E proerythroblasts; GEO GSE29196) and GATA1 (in differentiated G1E-ER-GATA1 cells; GEO GSE30142) occupancy and ATAC-seq profiles (in differentiated G1E-ER-GATA1 cells; GEO GSE114996) for each gene [38–40]. For the GATA2-upregulated genes, GATA2-occupied 21 of 27 SLCs. GATA1 occupied 17 of these genes. Accessible chromatin was detected at 24 of the 27 SLCs, coinciding with GATA1 and/or GATA2 occupancy. Of the 17 GATA2/GATA1-occupied genes, we compared the locations of the ChIP-seq peaks with respect to the genes, and 8 of the 17 SLCs harbored a WGATAR-containing predicted intronic enhancer. This cohort includes the equilibrative nucleoside transporter *Slc29a1*, magnesium transporter *Slc41a3*, cationic amino acid transporter *Slc7a1*, divalent metal ion transporter *Slc11a2*, anion exchanger *Slc4a1*, nucleobase transporter *Slc43a3*, branched-chain amino acid transporter *Slc25a44*, and *Slc22a23* with unknown function. Related SLC family members *Slc7a12*, *Slc22a28* and *Slc22a29* lacked ATAC-seq, GATA2 and GATA1 ChIP-seq peaks and served as negative controls (Fig 4B). These SLC genes, including *Slc29a1*, shared intronic GATA1 and GATA2 occupancy [38] that overlaps with accessible chromatin identified by our prior ATAC-seq analysis [39] and candidate *cis*-regulatory elements (cCREs) [41], providing evidence for GATA factor-mediated transcriptional regulation through the intronic sequences.

The GATA2 occupancy peaks of the eight SLC genes contained multiple WGATAR and E-Box motifs (Fig 4B). We asked if these motifs are enriched in these intronic sequences in comparison to randomly selected intronic sequences. The WGATAR and E-box motifs were enriched in the SLC intronic sequences relative to random intronic regions with the same length and a similar chromosomal location (*p* = 0.0054 and *p* = 0.0169, respectively). We asked if the regulatory attributes of this cohort are conserved in human. Four out of eight genes contain DNaseI hypersensitive sites (HSs) in human myeloid/erythroid cells [42] that overlap with GATA2 occupancy in K562 cells (GEO GSE18829) (S4 Fig). These sites are located in the same intron as the predicted murine enhancers, suggesting that the GATA factor regulatory mechanism may be conserved.

Gene expression analysis of the eight SLCs in erythroid precursors lacking the *Gata2*–77 enhancer revealed >1.5 fold reduced expression in mutant vs. wild type cells (Fig 4C). Thus, GATA2-activated these genes. Using our G1E-ER-GATA1 cell RNA-seq data [27], GATA1 repressed *Slc29a1*, *Slc41a3*, *Slc7a1*, and *Slc11a2* and activated *Slc4a1*, *Slc43a3*, *Slc25a44*, and *Slc22a23* (Fig 4D). *Slc7a12*, *Slc22a28*, and *Slc22a29* were not expressed in these systems. As these GATA1- and GATA2-regulated SLCs contained predicted intronic GATA1/2-occupied enhancers enriched in WGATAR and/or E-box motifs, we propose that these are direct GATA factor targets (Fig 4A).

### *Slc29a1* promotes erythroblast survival and differentiation *ex vivo*

**Fig 5:**
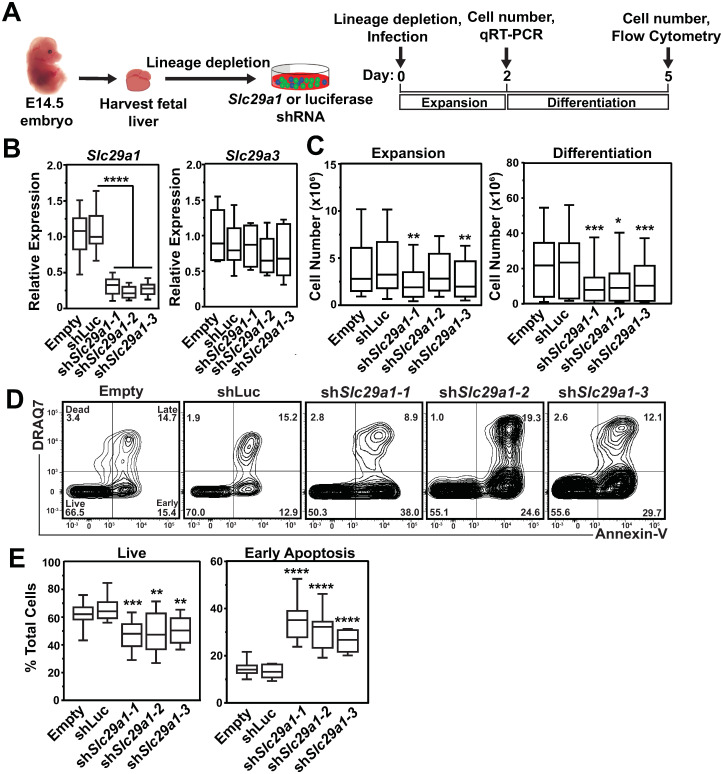
Slc29a1 confers survival during erythroblast differentiation. (A) Experimental strategy. HSPCs from E14.5 fetal livers were infected with *Slc29a1* or luciferase shRNAs. Erythroid precursors were expanded for two days followed by three days of erythroid differentiation. (B) qRT-PCR analysis validating *Slc29a1* mRNA knockdown efficiency. qRT-PCR analysis of *Slc29a1* and *Slc29a3* mRNAs. (C) Quantification of live cells after two days of erythroid precursor expansion (left). Quantification of live cells after two days of erythroid precursor expansion followed by three days of erythroid differentiation (right). (D) Flow cytometric analysis of Annexin V and the membrane-impermeable dye DRAQ7 in empty vector, luciferase or shRNA targeting *Slc29a1* during erythrocyte differentiation. (E) Quantification of cells in live and early apoptosis flow cytometric gates. *n* = 12 per condition (4 independent experiments). For the box-and-whisker plots, the box depicts the 25 to 75th percentiles of data, the median line, and whiskers ranging from minimum to maximum values. Statistical analysis was carried out using an unpaired 2-tailed Student’s *t* test to compare *Slc29a1* shRNA- and luciferase shRNA-infected cells. * *p* < 0.05, ** *p* < 0.01, *** *p* < 0.001, **** *p* < 0.0001.

Although GATA1 and GATA2 regulation of *Slc29a1* suggested that Slc29a1 has an important role in GATA factor regulation of hematopoiesis, equally possible was that it functions redundantly with other nucleoside-transporting SLCs. We isolated HSPCs from E14.5 murine fetal livers and used multiple distinct shRNAs to downregulate *Slc29a1* expression in a culture system that supports erythroid precursor expansion or differentiation. With or without *Slc29a1* downregulation, erythroid precursors were expanded for two days, GFP-positive cells expressing shRNAs were isolated by flow cytometry, and mRNA was quantified (Fig 5A). The shRNAs efficiently reduced *Slc29a1* mRNA without affecting mRNA for *Slc29a3* encoding the equilibrative nucleoside transporter ENT3 (Fig 5B). Under culture conditions that favor expansion over differentiation, *Slc29a1* downregulation reduced cell numbers in two of the three *Slc29a1*-targeting shRNAs relative to *luciferase* control shRNA-infected cells (37 ± 3.5% decrease, *p* = 0.037). After expanding cells for two days, erythroid precursors were transferred to erythropoietin-containing differentiation media for three days. Under differentiation conditions, *Slc29a1* downregulation decreased cell numbers relative to *luciferase* control shRNA-infected cells (56 ± 7.6% decrease, *p* = 0.0025) (Fig 5C). To investigate the mechanisms, erythroid precursors were expanded for two days and differentiated for three days. Using flow cytometry with apoptotic markers (Annexin V and DRAQ7), we quantified apoptosis in *Slc29a1* shRNA and control-infected primary erythroblasts (Fig 5D). *Slc29a1* downregulation increased apoptosis relative to cells expressing *luciferase* shRNA (61 ± 2.1% increase, *p* < 0.0001) (Fig 5E), indicating that Slc29a1 promotes erythroblast survival during erythroid differentiation.

**Fig 6:**
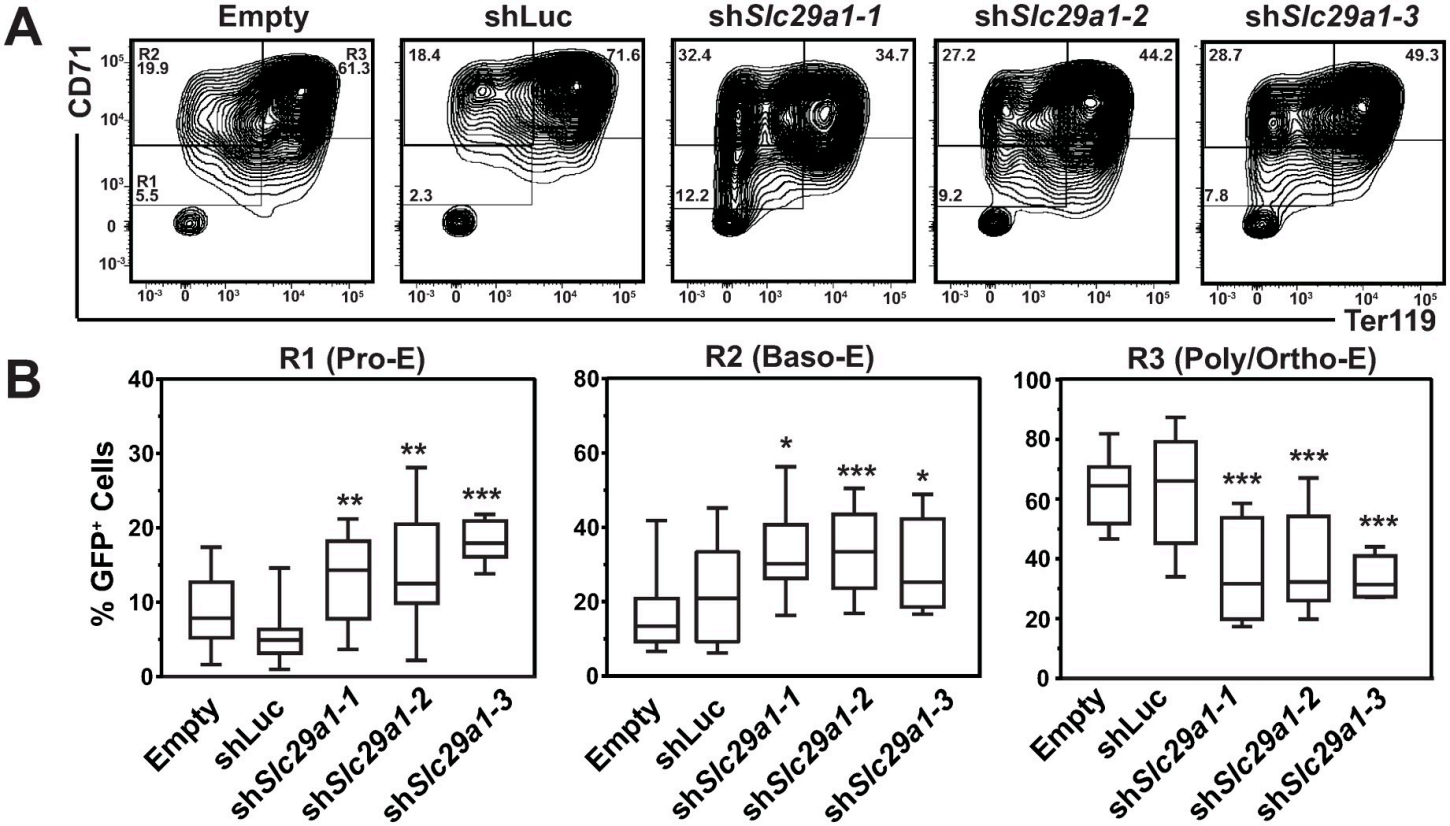
Slc29a1 promotes erythroid differentiation. (A) Flow cytometric analysis of erythroid markers CD71 and Ter119 in live GFP^+^-gated control and *Slc29a1*-shRNA infected erythroid precursor cells expanded for two days followed by three days of erythroid differentiation. (B) Quantification of the percentage of live GFP+-gated control and *Slc29a1*-shRNA infected conditions from R1-R3 gates (R1; proerythroblasts, R2; basophilic erythroblasts, R3; poly/orthochromatic erythroblasts). *n* = 12 per condition. Data are from 4 independent experiments. For the box-and-whisker plots, the box depicts the 25th to 75th percentiles of data, the median line, and whiskers ranging from minimum to maximum values. Statistical analysis was carried out using an unpaired 2-tailed Student’s *t* test to compare *Slc29a1* shRNA- and luciferase shRNA-infected cells. * *p* < 0.05, ** *p* < 0.01, *** *p* < 0.001, **** *p* < 0.0001.

Given the propensity for Slc29a1-deficient erythroblasts to undergo apoptosis, one would predict that this would impede the capacity of precursors to differentiate. Erythroid precursors were expanded for two days, followed by three days of differentiation to test whether *Slc29a1* downregulation influences erythroid differentiation (Fig 6A). Slc29a1 downregulation decreased poly/orthochromatic erythroblasts (R3) in comparison to cells expressing *luciferase* shRNA (54 ± 3.6% decrease, *p* = 0.0002) (Fig 6B). Proerythroblast (R1) and basophilic (R2) populations increased (58 ± 4.6% decrease in R1/R2, *p* = 0.0023). Thus, *Slc29a1* promotes erythroblast survival and erythroid differentiation.

### Genetic ablation of *Slc29a1* attenuates steady-state erythropoiesis and erythrocyte regeneration in response to acute anemia

**Fig 7:**
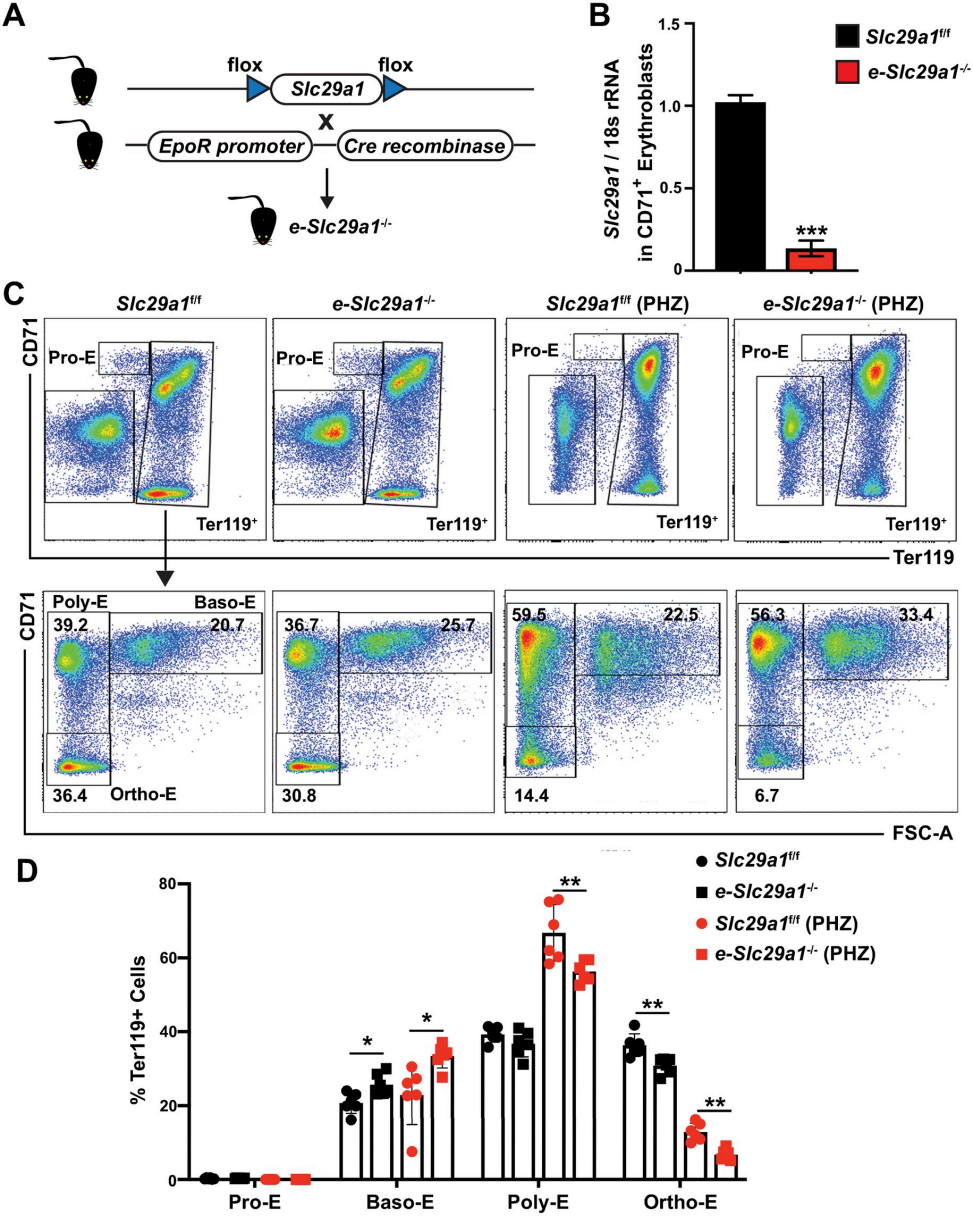
Targeted ablation of *Slc29a1* in erythroblasts attenuates steady-state erythropoiesis and erythrocyte regeneration in bone marrow of an acute anemia mouse model. (A) Generation of *e-Slc29a1*^-/-^ mice. (B) The relative expression of *Slc29a1* in CD71^+^ cells from *e-Slc29a1*^-/-^ and *Slc29a1*^f/f^ mice was quantified by qRT-PCR (normalized to 18s rRNA). Students’ *t* test, ***p < 0.001. (C) Representative image of Pro-E, Baso-E, Poly-E, and Ortho-E. Top panel: Pro-erythroblasts (Pro-E) represent CD71^high^Ter119^-^ population; Lower panel: In gated Ter119^+^ erythroblasts, based on CD71 and Ter119 expression level, coupled with forward scatter (FSC), three stages of erythroid differentiation were defined: basophilic erythroblasts (Baso-E) were Ter119^+^CD71^+^ with a greater FSC; polychromatic erythroblasts (Poly-E) were Ter119^+^CD71^+^ with reduced FSC; and orthochromatic erythroblasts (Ortho-E) were Ter119^+^CD71^-^. (D) Frequencies of erythroblast populations. Each dot represents data obtained from one mouse. Data were analyzed with a two-way ANOVA, followed by a Tukey’s multiple comparisons test, *p < 0.05, **p < 0.01.

Based on the Slc29a1 activity to promote survival and differentiation of primary fetal liver progenitor cells *ex vivo*, we evaluated whether these findings can be extrapolated *in vivo*. We bred floxed-*Slc29a1* mice with *EpoR*-Cre mice to specifically ablate Slc29a1 in erythroid cells (*e-Slc29a1*^-/-^) (Fig 7A). qRT-PCR analyses revealed that *Slc29a1* expression was specifically ablated in CD71^+^ erythroblasts isolated from bone marrow of *e-Slc29a1*^-/-^ mice (Fig 7B). The *e-Slc29a1*^-/-^ mice were fertile and had no obvious abnormalities. We compared hematological parameters by analyzing complete blood counts (CBC) of adult e-*Slc29a1*^-/-^ and *Slc29a1*^f/f^ mice. e-*Slc29a1*^-/-^ mice exhibited normal total Hb and HCT, slightly lower red blood cell (RBC) number (p < 0.05), and mildly increased MCV and MCH (p < 0.001 and p < 0.01, respectively), yet within the normal range relative to *Slc29a1*^f/f^ mice (Table 1).

**Table 1:** Hematological parameters of Slc29a1^f/f^ and e-Slc29a1^-/-^ mice treated with or without phenylhydrazine (PHZ).

	*Slc29a1*^*f/f*^ (untreated, n = 6)	*e-Slc29a1*^*-/-*^ (untreated, n = 9)	*Slc29a1*^*f/f*^ (PHZ, n = 8)	*e-Slc29a1*^*-/-*^ (PHZ, n = 7)
RBC (1x10 cells μl^-1^)	8.77 ± 0.49	7.91 ± 0.55*	5.90 ± 0.90***	4.88 ± 0.55*
Hb (g/dl^-1^)	12.76 ± 0.55	12.54 ± 1.09	10.50 ± 0.56**	8.32 ± 0.96***
HCT (%)	42.71 ± 3.39	41.14 ± 2.79	41.78 ± 3.04	34.92 ± 6.06*
MCV (fl)	47.50 ± 0.94	51.0 ± 2.27***	64.0 ± 3.71***	68.72 ± 2.2*
MCH (pg)	14.55 ± 0.43	15.53 ± 0.33**	17.07 ± 1.12***	18.75 ± 1.40*
WBC (1x10 cells μl^-1^)	10.79 ± 1.89	7.85 ± 4.09	7.09 ± 1.78	5.85 ± 3.05

*p <0.05,

**p<0.01,

***p<0.001,

12-week old *Slc29a1*^*f/f*^ and *e-Slc29a1*^*-/-*^ with equal number male and female mice were used. Mice were treated for six days with PHZ. Untreated *Slc29a1*^*f/f*^ mice were compared with untreated *e-Slc29a1*^*-/-*^. PHZ-treated *Slc29a1*^*f/f*^ were compared with PHZ-treated *e-Slc29a1*^*-/-*^ mice. PHZ-treated *Slc29a1*^*f/f*^ were compared with untreated *Slc29a1*^*f/f*^ mice.

In acute anemia, erythrocytes decline rapidly, and stress erythropoiesis is deployed to regenerate erythrocytes to attenuate the anemia. To determine the importance of erythroid Slc29a1 in acute anemia, we utilized a phenylhydrazine (PHZ)-induced hemolytic anemia model with elevated stress erythropoiesis. 12-week old *e*-*Slc29a1*^-/-^ mice and *Slc29a1*^f/f^ mice were injected consecutively with PHZ, and CBC analysis confirmed the PHZ-induced anemia in *Slc29a1*^f/f^ mice on day six. RBCs and total Hb decreased significantly (*p* < 0.001 and *p* < 0.01, respectively), while MCV and MCH increased (*p* < 0.001) (Table 1). However, PHZ-treated *e*-*Slc29a1*^-/-^ mice developed more severe anemia relative to PHZ-treated *Slc29a1*^f/f^ mice, exhibiting decreased RBCs (*p* < 0.05), total Hb (*p* < 0.001) and HCT (p < 0.05), and increased MCV and MCH (*p* < 0.05) (Table 1). This analysis provided *in vivo* genetic evidence that erythroid Slc29a1 protects against PHZ-induced acute anemia.

Because erythroid Slc29a1 is required for steady-state erythroid differentiation *ex vivo* and erythrocyte regeneration following PHZ treatment *in vivo*, we asked if Slc29a1 controls erythrocyte differentiation *in vivo*. Using flow cytometry with erythroid-specific surface markers Ter119 and CD71, we assessed differentiation in the adult bone marrow (BM). Without PHZ treatment, steady-state erythroid differentiation was mildly attenuated at the basophilic erythroblast (Baso-E; *p* < 0.05) to orthochromatic erythroblast (Ortho-E; *p* < 0.01) transition in bone marrow of e-*Slc29a1*^*-/-*^ mice relative to *Slc29a1*^*f/f*^ mice (Fig 7C–7D). PHZ-treated *e*-*Slc29a1*^-/-^ mice exhibited an attenuated transition from basophilic erythroblasts (Baso-E; *p* < 0.05) to polychromatic erythroblasts (Poly-E; *p* < 0.01) and orthochromatic erythroblasts (Ortho-E; *p* < 0.01), compared to PHZ-treated *Slc29a1*^f/f^ mice (Fig 7C–7D).

**Fig 8:**
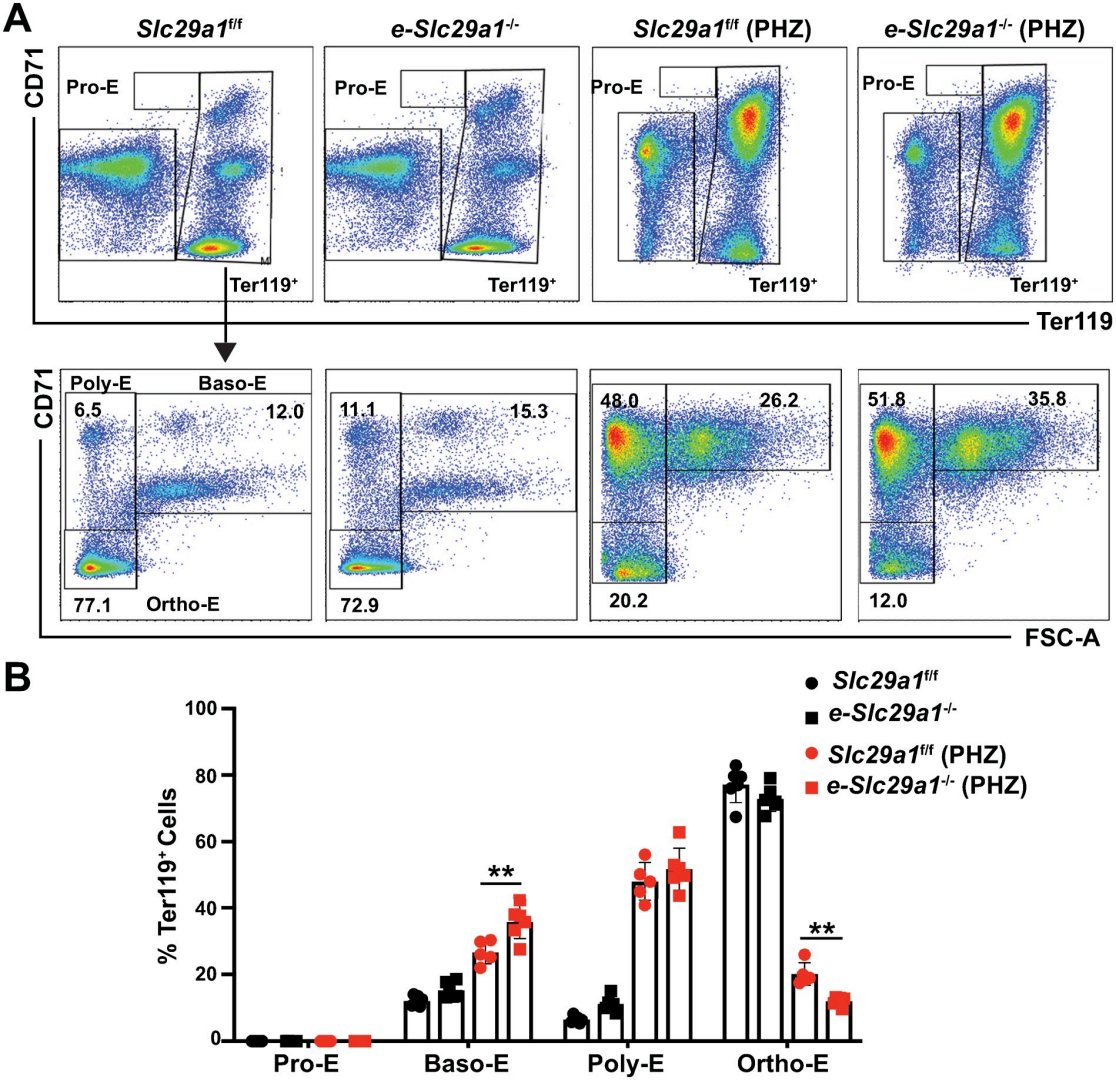
Targeted ablation of *Slc29a1* in erythroblasts attenuates erythrocyte regeneration in spleen of an acute anemia mouse model. (A) Representative image of Pro-E, Baso-E, Poly-E, Ortho-E (representing pro-erythroblasts, basophilic erythroblasts, polychromatic erythroblasts and orthochromatic erythroblasts) in spleen from *e-Slc29a1*^*-/-*^ and *Slc29a1*^f/f^ mice. Top panel: Pro-erythroblasts (Pro-E) represent the CD71^high^Ter119^-^ population; Lower panel: In gated Ter119^+^ erythroblasts, based on CD71 and Ter119 expression level, coupled with analysis of forward scatter (FSC), three stages of erythroid differentiation were defined: basophilic erythroblasts (Baso-E) were Ter119^+^CD71^+^ with a greater FSC; polychromatic erythroblasts (Poly-E) were Ter119^+^CD71^+^ with a reduced FSC; and orthochromatic erythroblasts (Ortho-E) were Ter119^+^ CD71^-^. (B) The frequencies of each erythroblast population. Data were analyzed with a two-way ANOVA, followed by a Tukey’s multiple comparisons test, *p < 0.05, **p < 0.01.

In mice, stress erythropoiesis occurs predominantly in the spleen [5, 43]. To test whether Slc29a1 counteracts PHZ-induced acute anemia in the spleen, we conducted flow cytometry to assess erythroid differentiation in *e*-*Slc29a1*^-/-^ and *Slc29a1*^f/f^ mouse spleen with or without PHZ treatment. Without PHZ, erythroid differentiation in *e*-*Slc29a1*^-/-^ and *Slc29a1*^f/f^ mouse spleen did not differ significantly (Fig 8A–8B). Similar to BM, following PHZ treatment, erythroid differentiation was greater in *Slc29a1*^f/f^ mice relative to *e-Slc29a1*^-/-^ mice, which exhibited an attenuated transition from Baso-E (*p* < 0.01) to Ortho-E (*p* < 0.01) (Fig 8A–8B). In aggregate, GATA factor-regulated Slc29a1 promoted cellular survival and erythroid differentiation *ex vivo*, and Slc29a1 promoted steady-state erythropoiesis and erythrocyte regeneration in bone marrow and spleen in an acute anemia model *in vivo*. These results position Slc29a1, along with a host of other GATA factor-regulated SLCs, as an important component of the GATA factor-dependent genetic network that governs erythrocyte development.

### Adenosine circuit embedded within GATA factor-regulated genetic networks

**Fig 9:**
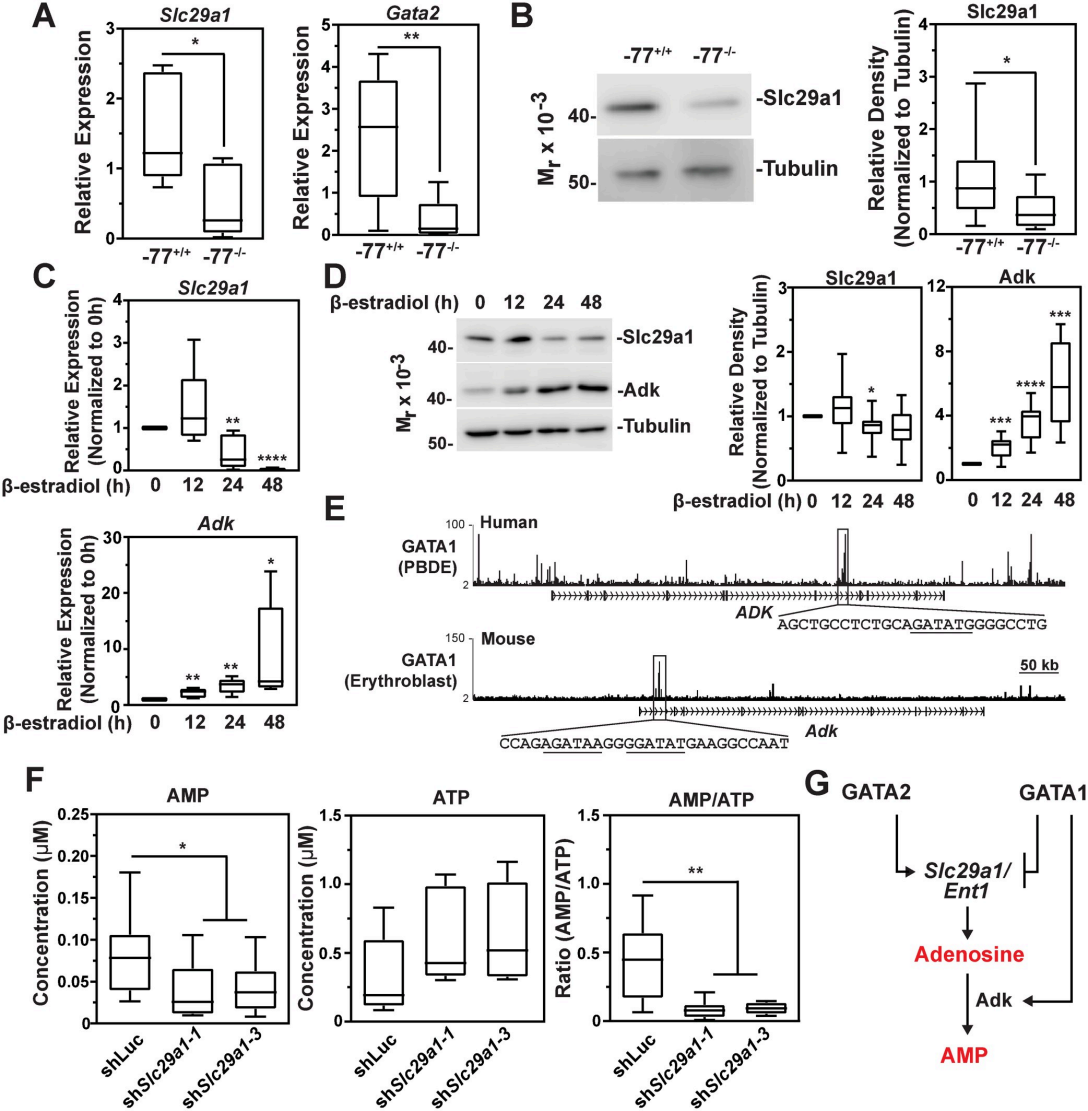
GATA factor regulatory circuit links Slc29a1 with adenosine mechanisms. (A) qRT-PCR analysis of *Slc29a1* (left) and *Gata2* (right) mRNA from *Gata2*–77^+/+^ and -77^-/-^ HSPCs isolated from E14.5 fetal liver (n = 10). (B) Representative Western blot analysis to detect Slc29a1 protein in *Gata2*–77^+/+^ and -77^-/-^ murine HSPCs isolated from E14.5 fetal liver (left). Quantification of relative band intensities from the immunoblots (n = 10; right). Tubulin served as a loading control. (C) qRT-PCR analysis of *Slc29a1* (top) or *Adk* (bottom) mRNA isolated from untreated vs β-estradiol-treated (12, 24, or 48 hours) G1E-ER-GATA1 cells. (D) Representative Western blot analysis to detect Slc29a1 and Adk protein levels in untreated vs β-estradiol-treated (12, 24, or 48 hours) G1E-ER-GATA1 cells (left). Quantification of relative band intensities from the immunoblots (right). Tubulin served as a loading control. Statistical significance was determined by comparing values at 12, 24, or 48-hour times to those from untreated cells (0 hour). Data are from 4 separate experiments (n = 9). (E) ChIP-seq profiles of GATA1 occupancy at human (GEO GSE32491; PBDE) *ADK* and murine (GEO GSE30142; Ter119+ erythroblast) *Adk* loci [40, 47]. The underlined sequences denote WGATAR motifs. (F) AMP and ATP concentrations quantified by a luciferase-based assay in primary erythroblasts infected with shRNA targeting luciferase or *Slc29a1* (left two panels). AMP/ATP ratio calculated from AMP and ATP concentrations (right). Data are from 4 separate experiments (n = 8). (G) Diagram depicting the relationships between GATA2, GATA1, *Slc29a1*, and *Adk*. For the box-and-whisker plots, the box depicts the 25th to 75th percentiles of data, the median line, and whiskers ranging from minimum to maximum values. Statistical analysis was carried out using an unpaired 2-tailed Student’s *t* test. * *p* < 0.05, ** *p* < 0.01, *** *p* < 0.001, **** *p* < 0.0001.

To further analyze the functional consequences of GATA factor-mediated *Slc29a1* transcription we analyzed the impact of GATA1 and GATA2 on Slc29a1 protein levels. Quantification of RNA and protein expression with -77^+/+^ or -77^-/-^ HSPCs isolated from E14.5 fetal livers revealed a significant reduction (68 ± 12%; p = 0.39) in *Slc29a1* mRNA in -77^-/-^ vs. -77^+/+^ cells (Fig 9A). As expected [30, 32], *Gata2* mRNA was significantly lower in -77^-/-^ vs. -77^+/+^ cells (87 ± 8.4%; p = 0.0026) (Fig 9A). To determine if GATA2 regulates Slc29a1 protein levels, we conducted semi-quantitative Western blotting in -77^+/+^ and -77^-/-^ E14.5 fetal liver-derived HSPCs. This analysis revealed a 48 ± 9.1% decrease (p = 0.043) in Slc29a1 protein in -77^-/-^ vs. -77^+/+^ cells. Thus, the GATA2-dependent transcriptional mechanism elevates Slc29a1 protein levels (Fig 9B).

To analyze GATA1 regulation, G1E-ER-GATA1 cells were treated with β-estradiol for various times, and qRT-PCR and semi-quantitative Western blotting was conducted 12, 24, and 48 hours following β-estradiol treatment. GATA1 decreased *Slc29a1* mRNA levels by 24 hours (59.7 ± 15.8%; p = 0.003) and at 48 hours (96.2 ± 1.2%; p < 0.0001). GATA1 decreased Slc29a1 protein by 24 hours (22.3 ± 4.9%; p = 0.023) (Fig 9C–9D).

Slc29a1 is the principle adenosine transporter in mammals. Another critical mechanism to control adenosine homeostasis involves the gene *Adk*, encoding adenosine kinase. Adenosine kinase phosphorylates adenosine to generate AMP, which can function as a signaling molecule to activate AMP-activated protein kinase (AMPK) [44] or act as a precursor molecule for important cellular processes. While targeted ablation of Adk causes neonatal hepatic steatosis [45] and vascular inflammation [46], Adk has not been studied in hematopoiesis.

RNA-seq data from G1E-ER-GATA1 cells [27] revealed GATA1 upregulation of *Adk* mRNA (S5A Fig). Quantitative proteomics data from G1E-ER-GATA1 cells [12] revealed GATA1-mediated upregulation of the two adenosine kinase isoforms >2-fold (S5B Fig). After β-estradiol treatment of G1E-ER-GATA1 cells for 12, 24 or 48 hours, *Adk* mRNA increased 2.2 ± 0.3-fold (p = 0.004), 3.5 ± 0.5-fold (p = 0.001) and 8.9 ± 3.5-fold (p = 0.04), respectively (Fig 9C). Similarly, GATA1 induced Adk protein 2.1 ± 0.2-fold (p = 0.0008), 3.6 ± 0.4-fold (p < 0.0001) and 5.9 ± 0.9-fold (p = 0.0001) at 12, 24 and 48 hours, respectively (Fig 9D). GATA1 occupied *ADK* intron 7 in primary human peripheral blood-derived erythroblasts (GEO GSE32491; PBDE) and intron 1 in primary murine erythroblasts (GEO GSE30142) [40, 47], with both intronic sites harboring GATA motifs (Fig 9E).

Since Adk catalyzes adenosine phosphorylation to yield AMP, and erythroid Slc29a1 loss decreases AMP levels and AMPK phosphorylation in mature erythrocytes [48], we asked if Slc29a1 downregulation decreases intracellular AMP in primary erythroblasts cultured *ex vivo* under conditions that promote erythroid cell expansion and differentiation. We isolated HSPCs from murine E14.5 fetal livers, infected them with two different shRNAs targeting *Slc29a1* or *luciferase*, and quantified AMP and ATP levels after sorting GFP-positive erythroblasts. This analysis revealed a 46 ± 7.6% (p = 0.033) decrease in AMP from cells expressing sh*Slc29a1* vs. *luciferase* shRNA. Although the ATP concentration did not differ significantly under these conditions, the AMP/ATP ratio was significantly lower (62 ± 3.5%; p = 0.004) in Slc29a1-deficient erythroblasts (Fig 9F), suggesting that the decreased AMP shifted cellular energy status. Thus, GATA factor regulation of Slc29a1 and Adk is associated with alterations in intracellular adenosine and AMP homeostasis.

In summary, GATA2 directly increased *Slc29a1* expression, and as GATA1 replaces GATA2 during erythroid differentiation, GATA1 repressed *Slc29a1*. GATA1 upregulated adenosine kinase, which controls metabolic processes, cellular signaling and adenosine homeostasis. Although it seems counterintuitive that GATA1 reduces Slc29a1 and elevates Adk, it is likely that multiple positive and negative regulatory components collectively control adenosine homeostasis. However, our discoveries elucidating this adenosine circuit embedded within GATA factor-instigated regulatory networks is proposed to be a critical link between GATA factor and adenosine mechanisms (Fig 9G).

## Discussion

**Fig 10:**
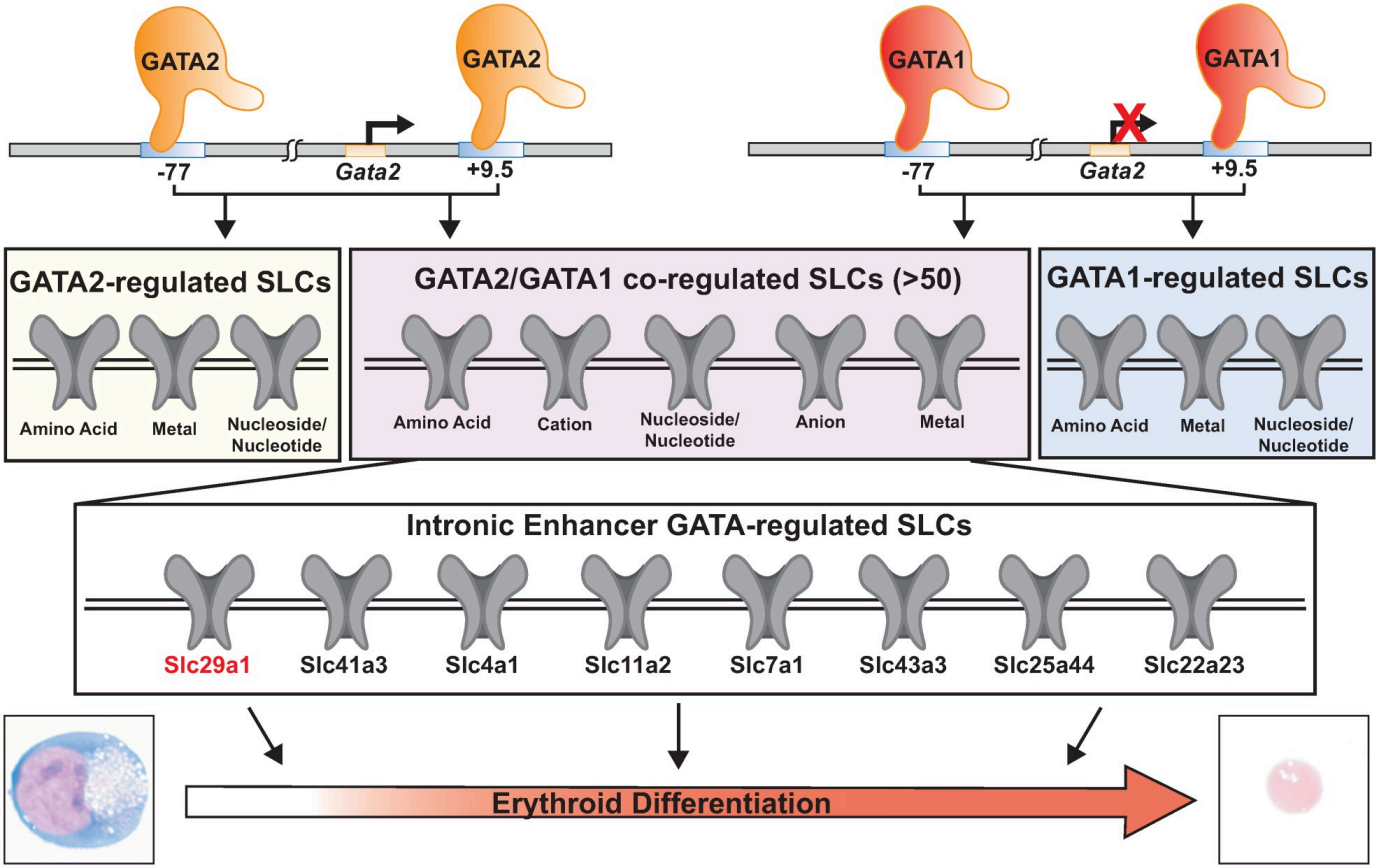
Model of GATA2 and GATA1 regulation of an SLC gene ensemble. Among the >50 GATA2- and GATA1-regulated SLCs, our studies with *Slc29a1*, *Slc41a3*, *Slc4a1*, *Slc11a2*, *Slc7a1*, *Slc43a3*, *Slc25a44*, and *Slc22a23* provided evidence for GATA2 and GATA1 occupancy and open chromatin at predicted intronic enhancers. Thus, this cohort may be regulated via a common mechanism. This GATA2- and GATA1-regulated ensemble is proposed to control diverse steps in erythrocyte development and regeneration.

Regulatory networks governing cell and developmental processes often include multiple members of large gene families. In a given biological context, numerous family members may share a common mechanism governing their expression and/or function, or distinct mechanisms may be committed to individual family members. Herein, we addressed this problem with the 456 member SLC transporter cohort. We demonstrated that GATA1 and GATA2 co-regulate >50 SLC genes, encoding amino acid, metal, nucleoside/nucleotide and additional SLC transporters (Fig 10). Our prioritization strategy identified a cohort of eight SLCs, including *Slc29a1*, that contain GATA1- and GATA2-occupied predicted intronic enhancers. Loss-of-function studies with Slc29a1, an equilibrative nucleoside transporter, indicated that Slc29a1 promotes erythroblast survival and differentiation in physiological and stress states by controlling intracellular adenosine homeostasis. By promoting erythroblast differentiation in spleen and BM in response to hemolytic anemia, erythroblast Slc29a1 mitigated anemia.

How does adenosine flux, sensing and consumption impact the generation of billions of erythrocytes daily? Adenosine is phosphorylated by adenosine kinase to yield AMP, which functions as a signaling molecule or precursor for ATP synthesis [46]. In an opposing pathway, adenosine deaminase-catalyzed conversion of adenosine to inosine is important for d*e novo* nucleotide biosynthesis to generate nucleic acids [49, 50]. AMP is a physiological activator of AMP-activated protein kinase [43], which inhibits mTOR signaling [51] and induces autophagy [52] that mediates intracellular remodeling required for erythroid differentiation [53, 54]. In addition, mTORC1 signaling controls mitochondrial biogenesis as a critical step in erythropoiesis [55]. GATA2 and GATA1 regulated Slc29a1 and adenosine kinase expression, thus constituting a GATA factor-adenosine circuit predicted to elevate AMP levels and stimulate AMP-dependent processes that impact erythrocyte development and regeneration. Nucleoside/nucleotide transporter involvement in GATA factor-instigated regulatory networks has not been described. Intracellular adenosine accumulation, e.g. in adenosine kinase deficiency, results in elevated s-adenosylhomocysteine, which inhibits s-adenosylmethionine (SAM)-dependent transmethylation reactions [32]. Since SAM is a requisite cofactor for DNA, RNA and protein methylation reactions that underlie epigenetic control of gene expression [56, 57], mechanisms governing adenosine homeostasis impact genome function.

The Slc29/ENT family consists of four members, with three mediating nucleoside transport and controlling a wide swath of cellular processes [58]. Slc29a1 transports adenosine with high affinity [36]. Previously, we demonstrated that targeted ablation of *Slc29a1* in erythroid cells elevates plasma adenosine to oppose acute hypoxia-induced tissue damage [59]. Furthermore, Slc29a1 downregulation at high altitude generates cytoprotective extracellular adenosine that mitigates hypoxia-mediated cell and tissue injury [59]. If sufficient erythrocyte regeneration does not occur, this injury can be lethal [5, 6]. Slc29a1 is also an important determinant of drug transport (e.g., ribavirin) into erythrocytes [60], suppresses mineralization of spinal tissues [61] and generates the “Augustine” blood group antigen [62]. However, its role in hematopoiesis and GATA factor mechanisms was not established. Slc29a3 (ENT3) also transports adenosine, but differs from Slc29a1 in its localization to intracellular compartments. *Slc29a3*^-/-^ mice exhibit HSC defects involving dysregulated autophagy and 5' AMP-Activated Protein Kinase activity [63]. Slc29a2/ENT2, which has high affinity for inosine [64], was GATA1-repressed.

As certain SLCs control nutrient availability and metabolic activity, mechanisms that unify or segregate actions of distinct SLC transporters and their respective small molecules are of considerable interest. These mechanisms may establish crucial links between extracellular stimuli, metabolic state and cellular transitions, including differentiation. Of the 31 known iron, zinc, and copper transporters, 22 were GATA factor-regulated, representing the majority of SLC metal transporters. These essential trace metals are important structural and functional co-factors in erythroid biology, with the best studied being iron-containing heme [65]. The initial and rate-limiting step of heme synthesis in the mitochondrial matrix requires succinyl-CoA, derived from the tricarboxylic acid cycle, and glycine, which can be acquired through SLC transport mechanisms [24, 66] or biosynthetic pathways.

In summary, we demonstrated that hematopoietic-regulatory GATA factors control a >50 SLC gene ensemble, and one member, Slc29a1, promotes erythroblast survival and erythroid differentiation. Transporter co-regulation may be a primary determinant, or one of multiple parameters, of an integrated network that dynamically controls the metabolome and metallome. It will be instructive to consider the mechanistic interconnections or independence of the transporters and their small molecule passengers in cellular survival, proliferation and differentiation, both in the context of hematopoiesis and broad biological contexts.

## Materials and methods

### Fetal liver hematopoietic stem/progenitor cell isolation

Lineage-negative (Lin^-^) HSPCs were isolated from wild type E14.5 C57BL6/J embryonic fetal livers as described [67]. Fetal livers were homogenized in PBS containing 2% fetal bovine serum (FBS), 2.5 mM EDTA and 10 mM glucose. The homogenate was incubated with biotin-conjugated anti-mouse CD3ε (3 μl/ml), anti-mouse CD11b (3 μl/ml), anti-mouse CD19 (3 μl/ml), anti-mouse CD45R (3 μl/ml), anti-mouse GR-1 (3 μl/ml), anti-mouse CD71 (3 μl/ml) and anti-mouse Ter119 (5 μl/ml) for 15 min at 4°C. Cells were washed once with phosphate-buffered saline (PBS) containing 2% FBS, 2.5 mM EDTA, and 10 mM glucose, followed by incubation with MojoSort Streptavidin Nanobeads (75 μl/ml) for 15 min at 4°C. Cells were washed by resuspension in 3 ml PBS containing 2% FBS, 2.5 mM EDTA and 10 mM glucose, followed by centrifugation at 300 x g for 5 min at 4°C. Cells were resuspended in 2.5 ml PBS containing 2% FBS, 2.5 mM EDTA and 10 mM glucose, incubated for 5 min with a magnet, and unbound cells containing HSPCs were collected.

### Cell culture

HSPCs were cultured in StemPro-34 containing 1X nutrient supplement (Thermo Fisher), 2 mM L-glutamine (Thermo Fisher), 1% penicillin/streptomycin (Thermo Fisher), 100 μM monothioglycerol (Sigma), 1 μM dexamethasone (Sigma), 0.5 U/ml erythropoietin, and 100 ng/ml recombinant SCF to expand erythroid precursor cells. After expanding cells for two days, the cells were cultured in IMDM containing 10% FBS, 10% Plasma-derived Serum (Animal Technologies), 5% Protein-free Hybridoma Medium II (Thermo Fisher), 2 mM L-glutamine, 1% penicillin/streptomycin, 100 μM monothioglycerol and 6 U/ml erythropoietin for three days to promote erythroid differentiation. Live cells were quantified using a Cellometer Auto T4 cell counter (Nexcelom Bioscience) after expanding cells for two days and then differentiating for three days.

### shRNA construction and retroviral infection

Three distinct miR-30 shRNAs targeting *Slc29a1* or luciferase as a control were cloned into MSCV-PIG vectors (IRES-GFP) using *Xho* I and *Bgl* II restriction sites [12, 68]. An empty MSCV-PIG vector was used as an additional negative control. MSCV-PIG vectors and pCL-Eco packaging vector (15 μg of each) were transfected into 293T cells to produce retrovirus targeting *Slc29a1* or *luciferase*. For shRNA-mediated loss-of-function experiments, 3 x 10^5^ HSPCs were spinoculated with 100 μl retrovirus, 8 μg/ml polybrene and 10 mM HEPES buffer at 1600 x g for 90 min at 30°C. After expanding cells for two days, live GFP^+^ cells were sorted from the cell population using a BD FACSAria cell sorter (BD Biosciences), and gene expression was quantified by qRT-PCR.

### Quantitative real-time PCR analysis

Total RNA was purified with TRIzol (Life Technologies). RNA (1 μg) was DNase treated for 15 min and then heated at 65°C for 10 min with 25 mM EDTA. For cDNA synthesis, DNase I-treated RNA was incubated with 125 ng of a 5:1 mixture of oligo-dT primers and random hexamers at 68°C for 10 min. The RNA and primer mixture was incubated with Moloney MLV reverse transcriptase (Life Technologies), 10 mM DTT, RNAsin (Promega), and 0.5 mM deoxynucleoside triphosphates at 42°C for 1 hour followed by heat inactivation at 98°C for 5 min. qRT-PCR was conducted with Power SYBER Green Master Mix (Applied Biosystems) and a ViiA 7 Real-Time PCR system (Applied Biosystems).

### Flow cytometry

For detection of erythroid markers, 1 x 10^6^ cells were isolated by centrifugation at 300 x g for 5 minutes at 4°C and washed once in PBS. Cells were incubated in 100 μl PBS with 1:100 anti-mouse Ter119-APC (Biolegend) and 1:100 anti-mouse CD71-PE (Biolegend) at 4°C for 30 min in the dark. To quantify apoptosis following CD71/Ter119 staining, cells were washed once in 1X Annexin V Buffer (10mM HEPES (Sigma), 140 mM NaCl, 2.5 mM CaCl_2_) and stained with 1:40 Annexin V-Pacific Blue (Thermo Fisher) and 1:100 membrane-impermeable dye DRAQ7 (Abcam) at room temperature for 20 minutes in the dark. Cells were isolated by centrifugation for 5 minutes, 300 x g at 4°C and resuspended in 300 μl PBS. Flow cytometry was conducted with a BD LSRII flow cytometer (BD Biosciences), and data was analyzed with FlowJo software (FlowJo).

Bone marrow and spleen cells were analyzed to quantify erythroid differentiation. Antibodies (Biolegend) were used at a concentration of 1:100 unless indicated. Bone marrow and spleen cells were stained with Pacific Blue-conjugated Ter119 and PE/CY7-conjugated CD71 for one hour or overnight on ice. Cells were centrifuged, resuspended and analyzed with a BD LSRII flow cytometer (BD Biosciences).

### Western blot analysis

Cells (1 x 10^6^) were isolated by centrifugation at 1300 x g for 5 min at 4°C, washed once in PBS, and lysed by boiling in Sodium Dodecyl Sulfate (SDS) sample buffer (50mM Tris, pH 6.8, 2% β-mercaptoethanol, 2% SDS, 0.04% bromophenol blue, 10% glycerol) for 10 minutes. Whole cell lysates were resolved by SDS-PAGE, and proteins were quantified using chemiluminescence detected with FEMTO supersignal (Pierce). Densitometry analysis was performed with Image Studio (LI-COR Biosciences). The following primary antibodies were used: 1:1000 anti-Slc29a1/ENT1 (Santa Cruz Biotechnology; sc-377283), 1:1000 anti-ADK (Abcam; ab227087), and 1:1000 anti-α-tubulin (ThermoFisher; 14-4502-82). HRP-conjugated anti-mouse and HRP-conjugated anti-rabbit secondary antibodies (Jackson Labs) were used.

### AMP and ATP quantification

Equal numbers of cells were isolated by FACS, followed by centrifugation at 1300 x g for 5 min at 4°C. Intracellular ATP concentration was quantified with Cell-Titer Glo Luminescent Cell Viability Assay (Promega) following manufacturers’ instructions. Intracellular AMP was quantified with AMP-Glo Assay (Promega) following manufacturers’ instructions. ATP and AMP analysis was conducted with a PheraStar Microplate Reader (BMG Labtech).

### Mice and phenylhydrazine (PHZ) studies

Animal protocols were reviewed and approved by the Institutional Animal Welfare Committee of UT Health Science Center at Houston. *Slc29a1*^*flox/flox*^ (*Slc29a1*^*f/f*^) were obtained from Dr. Holger Eltzschig (UT Health Science Center at Houston). *EpoR-Cre*^*+*^ mice were obtained from Dr. Stuart Orkin (Harvard Medical School). *Slc29a1*^*f/f*^ *EpoR-Cre*^*+*^ mice (*e-Scl29a1*^*-/-*^) were generated by crossing *Slc29a1*^*f/f*^ with *EpoR-Cre*^*+*^ mice and genotyped as *Slc29a1*^*f/f +/+*^ *EpoR-Cre*^*+*^ [57]. Twelve-week-old (sex-matched, male and female) *Slc29a1*^*f/f*^ and e-*Slc29a1*^*-/-*^ mice were used for experiments. *Slc29a1*^*f/f*^ and *e-Slc29a1*^*-/-*^ mice were treated with vehicle or PHZ (Sigma Aldrich, P26252) at 50 mg/kg on day 0 and day 1 by intraperitoneal injection. At day 7, mice were sacrificed, and blood was collected for complete blood count (CBC) analysis.

### CD71^+^ erythroblast purification and analysis of *Slc29a1* ablation in erythroblasts

CD71^+^ erythroblasts were purified from bone marrow by CD71 monoclonal antibody (Santa Cruz, sc-59112) conjugated to Dynabeads (Invitrogen, 11033) following manufacturers’ instructions. Anti-CD71 was incubated with Dynabeads on ice for 30–60 min and washed twice with Hank’s Balanced Salt Solution (HBSS) buffer (Gibco, 14025076). Bone marrow cells were incubated with Dynabead-conjugated antibodies on ice for 60 min, purified by magnetic separation and cells were washed twice with HBSS buffer. mRNA was isolated, converted to cDNA and *Slc29a1* mRNA levels were quantified with the following primers: mouse *18S*, forward 5′-GTAACCCGTTGAACCCCATT-3′ and reverse 5′-CCATCCAATCGGTAGTAGCG-3′; mouse *Slc29a1*, 5′-CTTGGGATTCAGGGTCAGAA-3′ and reverse 5′-ATCAGGTCACACGACACCAA-3′.

### Statistical analysis

Statistical analysis for *ex vivo* studies was conducted with the 2-tailed Student’s *t* test to compare experimental and control samples. Asterisks denote statistical significance relative to controls. For *in vivo* studies, all data are expressed as the mean ± SD. Differences between the means of multiple groups were compared by two-way ANOVA, followed by a Tukey’s multiple comparisons test. P < 0.05 was considered significant. Statistical significance was analyzed with GraphPad Prism 8 software (GraphPad).

Motif enrichment analysis for WGATAR and/or E-Box motifs was conducted by generating eight comparable sequences to those observed from the intronic enhancer regions for the eight SLC genes regulated by both GATA2- and GATA1. 10,000 groups of eight sequences were randomly generated, and the proportion of times out of 10,000 in which the observed intronic enhancer sequences contained a WGATAR and/or E-Box motif was used to calculate statistical significance (p-value). This enrichment analysis was carried out with R software.

**Key points**

- GATA factors regulate a small molecule transporter cohort.
- Nucleoside transporter Slc29a1 promotes erythroblast survival, differentiation, and regeneration to surmount anemia.
- GATA factor-Slc29a1 circuit controls adenosine homeostasis.

## Supporting information

S1 Fig GATA1-regulated amino acid and metal SLC transporter cohorts. (A) The heatmap depicts GATA1-regulated amino acid SLC and the thiamine transporter *Slc19a2* mRNAs derived from comparison of G1E-ER-GATA1 cell RNA-seq data (untreated vs. 48 hour β-estradiol-treated cells) (AA, amino acid; BCAA, branched-chain amino acid). (B) The heatmap depicts GATA2-regulated amino acid SLC mRNAs from comparison of *Gata2*–77^-/-^ vs. wild-type erythroid precursor RNA-seq data. (B) Schematic representation of established substrates and cellular/subcellular localizations of the GATA2-regulated amino acid SLC transporter cohort. (C) The heatmap depicts GATA1-regulated metal SLC mRNAs derived from comparison of G1E-ER-GATA1 cell RNA-seq data (untreated vs. 48 hour β-estradiol-treated cells). (D) The heatmap depicts GATA2-regulated metal SLC mRNAs from comparison of *Gata2*–77^+/+^ vs. -77^-/-^ erythroid precursor RNA-seq data.
(TIF)

S2 Fig Functional annotation of GATA factor-regulated SLCs. (A) The pie charts depict the percentages of the amino acid transporter cohorts represented in the 456 member SLC ensemble, 194 SLCs expressed in the G1E-ER-GATA1 system, 277 SLCs expressed in *Gata2*–77^+/+^ primary murine erythroblasts, 112 SLCs regulated by GATA1, but not GATA2, and 42 SLCs regulated by GATA2, but not GATA1, and 53 GATA1/GATA2-co-regulated SLCs. (B) The pie charts depict the percentages of the metal transporter cohort within the 456 member SLC ensemble, 194 SLCs expressed in G1E-ER-GATA1 cells, 277 SLCs expressed in *Gata2*–77^+/+^ primary erythroblasts, 112 SLCs regulated by GATA1, but not GATA2, and 42 SLCs regulated by GATA2, but not GATA1, and 53 GATA1/GATA2-co-regulated SLCs. (C) The pie charts depict the percentages of the nucleoside/nucleotide transporter cohort within the 456 member SLC ensemble, 194 SLCs expressed in G1E-ER-GATA1 cells, 277 SLCs expressed in *Gata2*–77^+/+^ erythroblasts, 112 SLCs regulated by GATA1, but not GATA2, and 42 SLCs regulated by GATA2, but not GATA1, and 53 GATA1/GATA2-co-regulated SLCs. Differences between the 456 member SLC ensemble and GATA factor-regulated SLC cohorts were determined with a Chi-Square test.
(TIF)

S3 Fig Differential expression of GATA2- and GATA1-regulated SLCs during human erythroid differentiation. The graphs were generated from RNA-seq data of gene expression during human CD34^+^ cell differentiation into erythrocytes [37] (GEO GSE53983). (A) GATA1-activated genes. *SLC4A1*, *SLC38A5* and *SLC28A3* expression during human erythroid differentiation. (B) GATA1-repressed genes. *SLC29A1*, *SLC41A3*, and *SLC16A1* expression during human erythroid differentiation. (C) GATA2-activated genes. *SLC25A21*, *SLC40A1* and *SLC22A23* expression during human erythroid differentiation. (D) GATA2-repressed genes. *SLC9A5*, *SLC12A9*, *and SLC43A2* expression during human erythroid differentiation. Pr, Proerythroblast; EB, Early basophilic erythroblast; LB, Late basophilic erythroblast; Po, Polychromatic erythroblast; O, Orthochromatic erythroblast. Bar graphs depict mean ± SEM. * *p* < 0.05, ** *p* < 0.01, *** *p* < 0.001, **** *p* < 0.0001.
(TIF)

S4 Fig Profiles of Human DNaseI HS Sites in a GATA2-regulated SLC Cohort. Profiles of DNaseI HS sites in human HSPCs (red) and vascular endothelial cells (black) [42]. Enhanced DNaseI HS region shows location of human K562 GATA2 ChIP-seq peak (GEO GSE18829) denoted by black bar.
(TIF)

S5 Fig GATA1 regulates adenosine kinase during erythroid maturation. (A) RNA-seq data comparing mRNA levels in -77^+/+^ vs. -77^-/-^ primary murine erythroid precursor cells (left) [31] and control vs. β-estradiol-treated (48 h) G1E-ER-GATA1 cells (right) [27]. The scatter plots represent means ± SEM. TPM; Transcripts per million (*n* = 3). (B) Quantitative proteomics data [12] illustrating GATA1 upregulation of adenosine kinase isoforms (± 48 hours of β-estradiol treatment of G1E-ER-GATA1).
(TIF)

S1 Table GATA2- and GATA1-regulated SLCs. (XLSX)
