# Supplementary figures and images for: GATA factor-regulated solute carrier ensemble reveals a nucleoside transporter-dependent differentiation mechanism
Source: PLoS Genet. 2020 Dec 28;16(12):e1009286. doi: 10.1371/journal.pgen.1009286 (PMC7793295; doi:10.1371/journal.pgen.1009286)

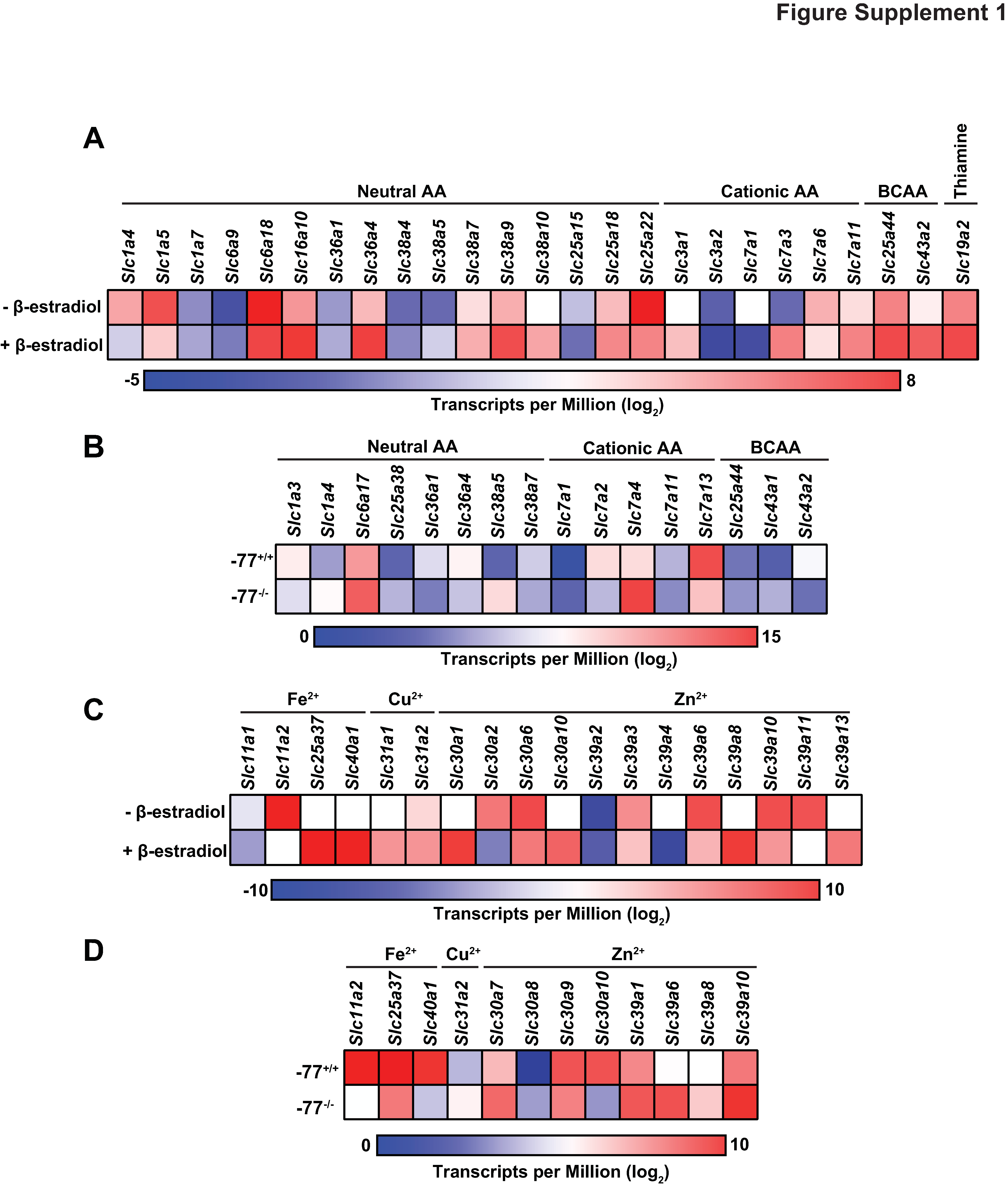

Supplement: S1 Fig — (A) The heatmap depicts GATA1-regulated amino acid SLC and the thiamine transporter Slc19a2 mRNAs derived from comparison of G1E-ER-GATA1 cell RNA-seq data (untreated vs. 48 hour β-estradiol-treated cells) (AA, amino acid; BCAA, branched-chain amino acid). (B) The heatmap depicts GATA2-regulated amino acid SLC mRNAs from comparison of Gata2–77-/- vs. wild-type erythroid precursor RNA-seq data. (B) Schematic representation of established substrates and cellular/subcellular localizations of the GATA2-regulated amino acid SLC transporter cohort. (C) The heatmap depicts GATA1-regulated metal SLC mRNAs derived from comparison of G1E-ER-GATA1 cell RNA-seq data (untreated vs. 48 hour β-estradiol-treated cells). (D) The heatmap depicts GATA2-regulated metal SLC mRNAs from comparison of Gata2–77+/+ vs. -77-/- erythroid precursor RNA-seq data. (TIF) [file pgen.1009286.s001.tif]

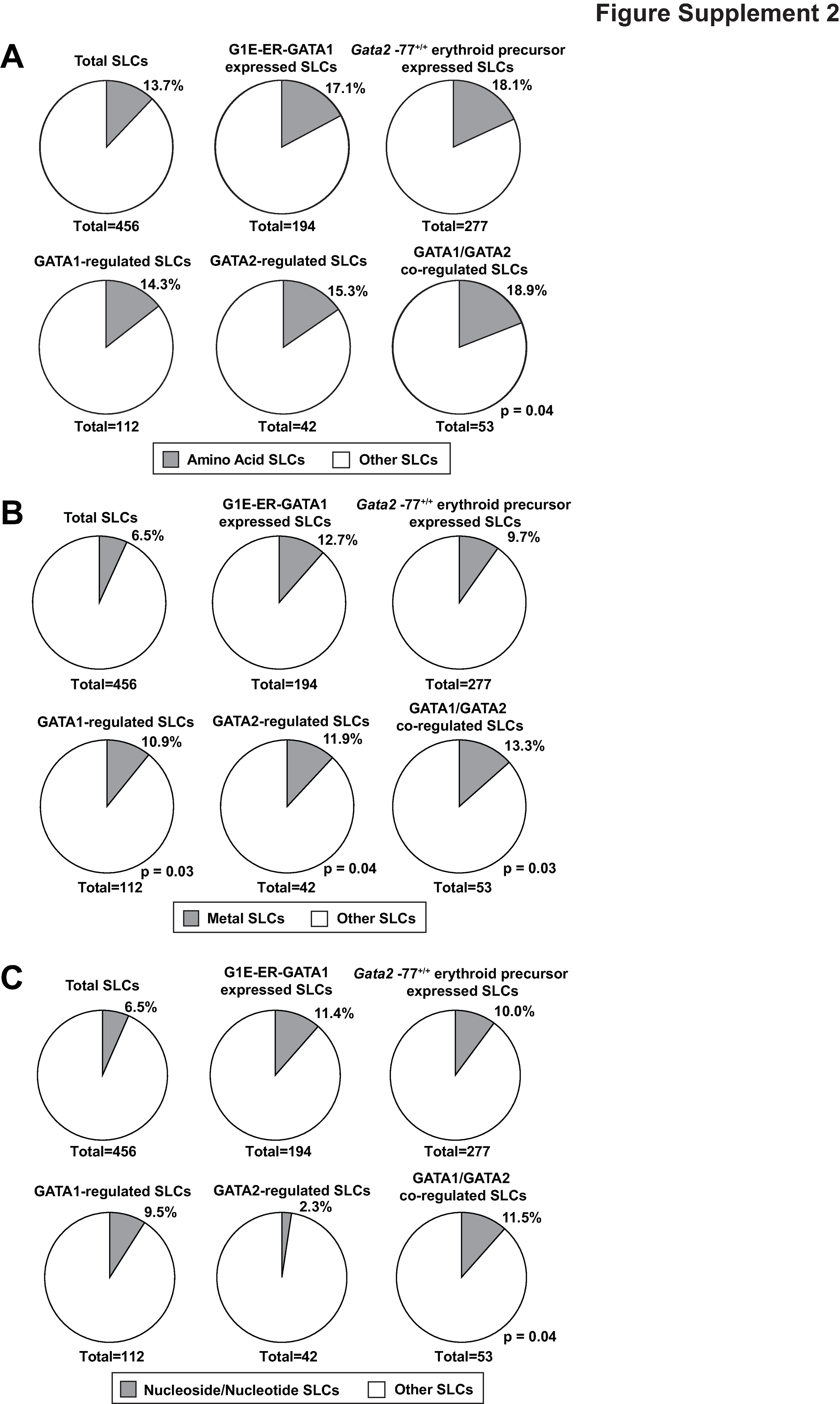

Supplement: S2 Fig — (A) The pie charts depict the percentages of the amino acid transporter cohorts represented in the 456 member SLC ensemble, 194 SLCs expressed in the G1E-ER-GATA1 system, 277 SLCs expressed in Gata2–77+/+ primary murine erythroblasts, 112 SLCs regulated by GATA1, but not GATA2, and 42 SLCs regulated by GATA2, but not GATA1, and 53 GATA1/GATA2-co-regulated SLCs. (B) The pie charts depict the percentages of the metal transporter cohort within the 456 member SLC ensemble, 194 SLCs expressed in G1E-ER-GATA1 cells, 277 SLCs expressed in Gata2–77+/+ primary erythroblasts, 112 SLCs regulated by GATA1, but not GATA2, and 42 SLCs regulated by GATA2, but not GATA1, and 53 GATA1/GATA2-co-regulated SLCs. (C) The pie charts depict the percentages of the nucleoside/nucleotide transporter cohort within the 456 member SLC ensemble, 194 SLCs expressed in G1E-ER-GATA1 cells, 277 SLCs expressed in Gata2–77+/+ erythroblasts, 112 SLCs regulated by GATA1, but not GATA2, and 42 SLCs regulated by GATA2, but not GATA1, and 53 GATA1/GATA2-co-regulated SLCs. Differences between the 456 member SLC ensemble and GATA factor-regulated SLC cohorts were determined with a Chi-Square test. (TIF) [file pgen.1009286.s002.tif]

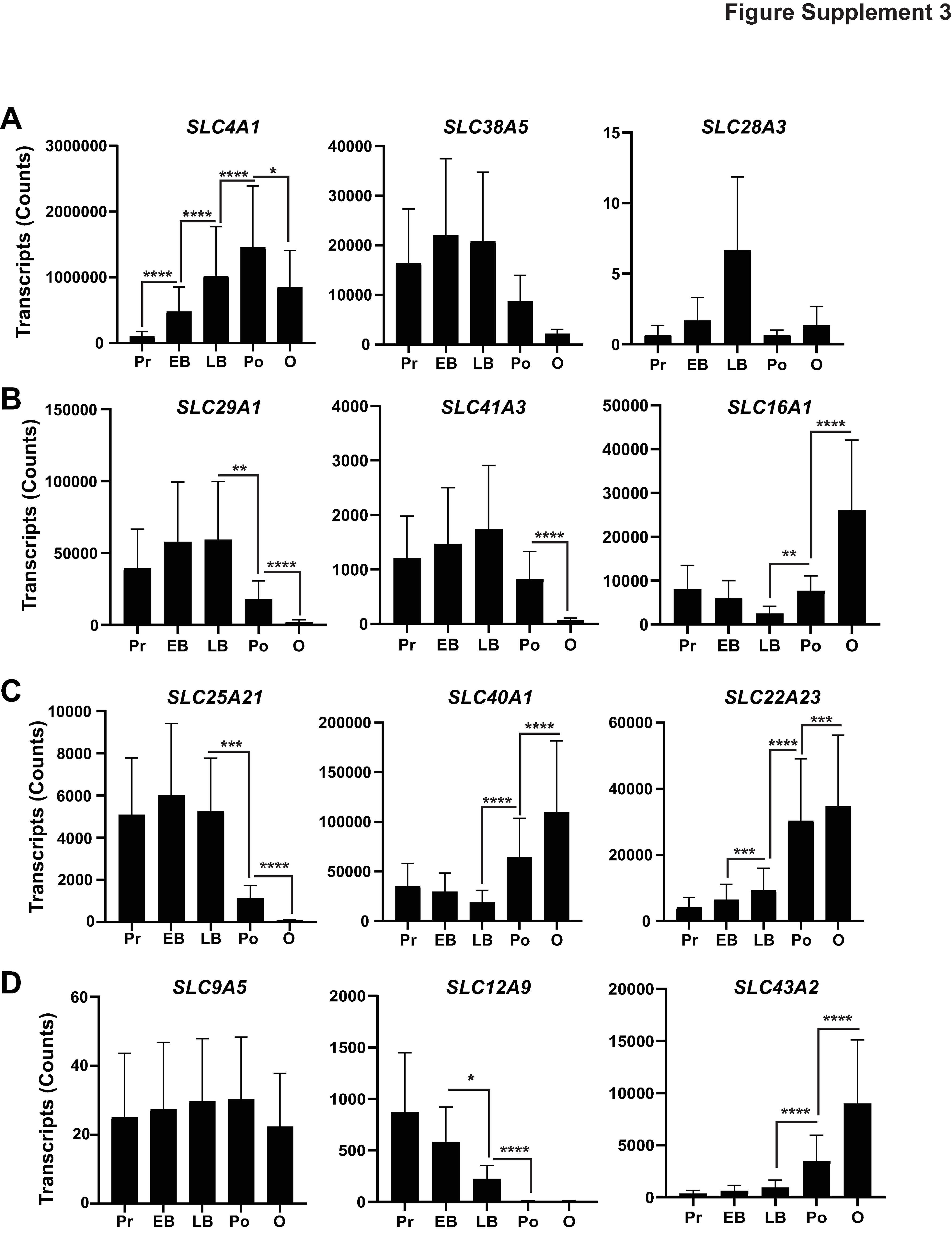

Supplement: S3 Fig — The graphs were generated from RNA-seq data of gene expression during human CD34+ cell differentiation into erythrocytes [37] (GEO GSE53983). (A) GATA1-activated genes. SLC4A1, SLC38A5 and SLC28A3 expression during human erythroid differentiation. (B) GATA1-repressed genes. SLC29A1, SLC41A3, and SLC16A1 expression during human erythroid differentiation. (C) GATA2-activated genes. SLC25A21, SLC40A1 and SLC22A23 expression during human erythroid differentiation. (D) GATA2-repressed genes. SLC9A5, SLC12A9, and SLC43A2 expression during human erythroid differentiation. Pr, Proerythroblast; EB, Early basophilic erythroblast; LB, Late basophilic erythroblast; Po, Polychromatic erythroblast; O, Orthochromatic erythroblast. Bar graphs depict mean ± SEM. * p < 0.05, ** p < 0.01, *** p < 0.001, **** p < 0.0001. (TIF) [file pgen.1009286.s003.tif]

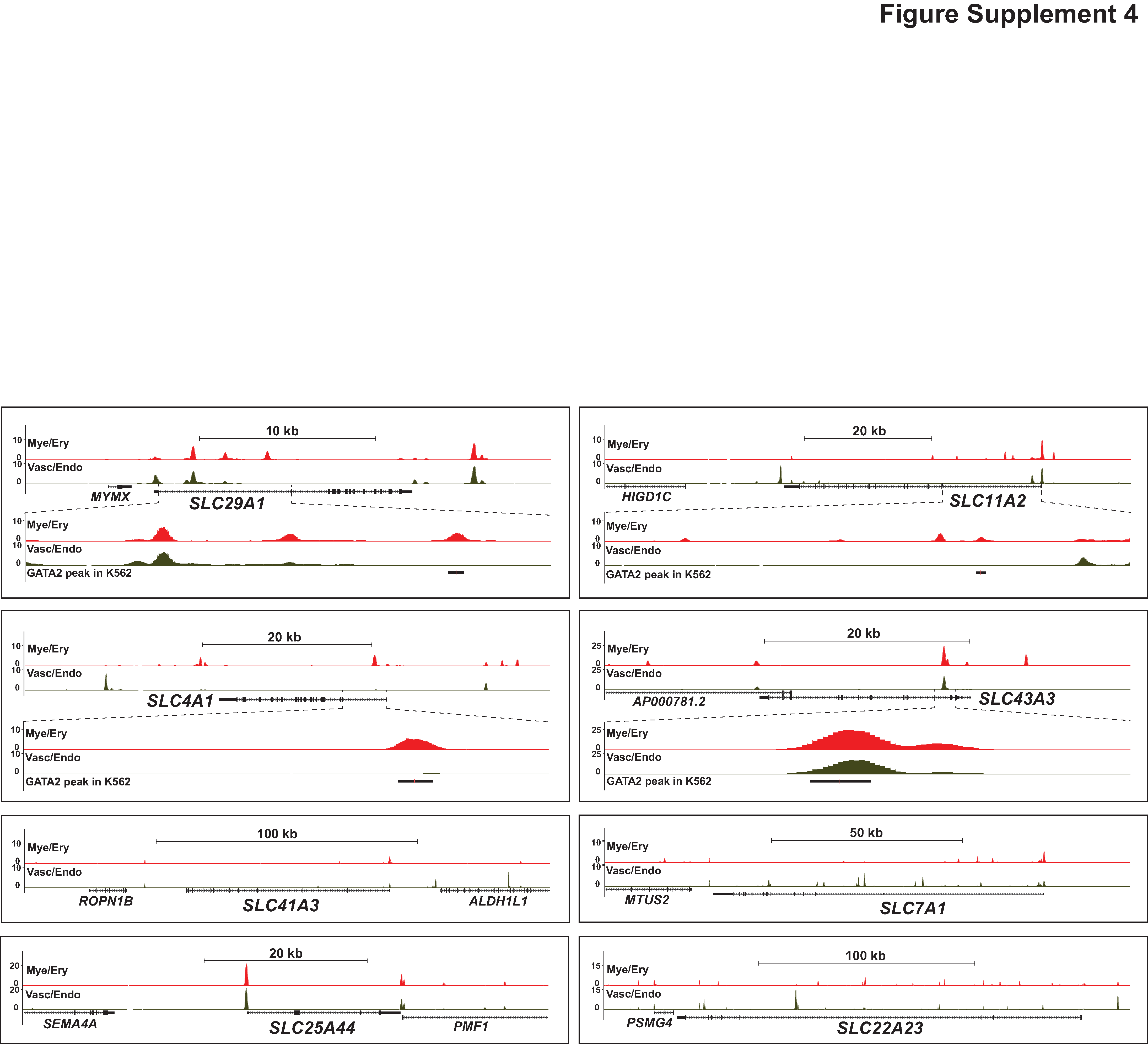

Supplement: S4 Fig — Profiles of DNaseI HS sites in human HSPCs (red) and vascular endothelial cells (black) [42]. Enhanced DNaseI HS region shows location of human K562 GATA2 ChIP-seq peak (GEO GSE18829) denoted by black bar. (TIF) [file pgen.1009286.s004.tif]

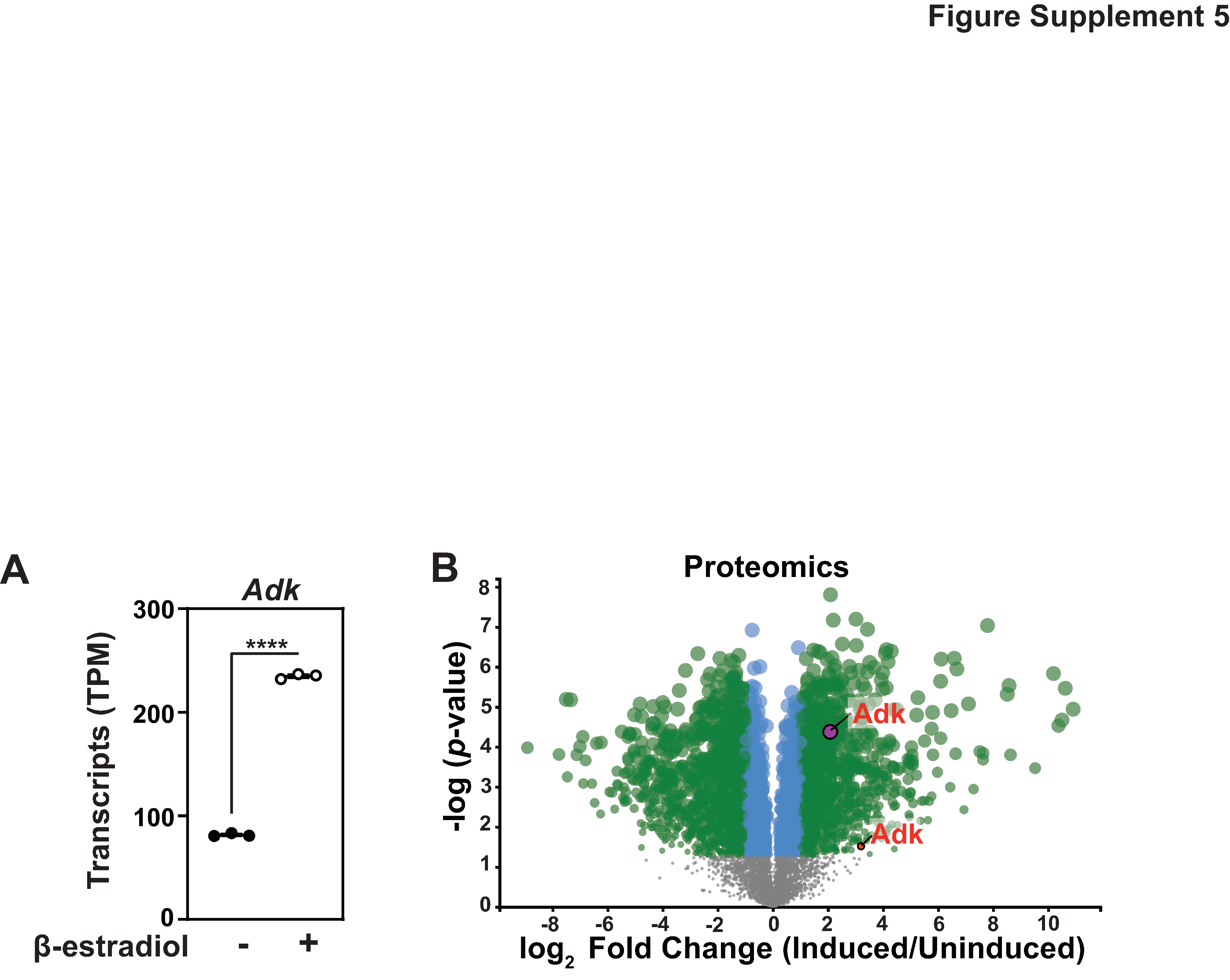

Supplement: S5 Fig — (A) RNA-seq data comparing mRNA levels in -77+/+ vs. -77-/- primary murine erythroid precursor cells (left) [31] and control vs. β-estradiol-treated (48 h) G1E-ER-GATA1 cells (right) [27]. The scatter plots represent means ± SEM. TPM; Transcripts per million (n = 3). (B) Quantitative proteomics data [12] illustrating GATA1 upregulation of adenosine kinase isoforms (± 48 hours of β-estradiol treatment of G1E-ER-GATA1). (TIF) [file pgen.1009286.s005.tif]
